# Clinical Trials in Cardiac Arrest and Subarachnoid Hemorrhage: Lessons from the Past and Ideas for the Future

**DOI:** 10.1155/2013/263974

**Published:** 2013-03-07

**Authors:** Jennifer A. Frontera

**Affiliations:** Cleveland Clinic Foundation, Neurological Institute, Cerebrovascular Center, Cleveland Clinic, 9500 Euclid Avenue S80, Cleveland, OH 44195, USA

## Abstract

*Introduction*. Elevated intracranial pressure that occurs at the time of cerebral aneurysm rupture can lead to inadequate cerebral blood flow, which may mimic the brain injury cascade that occurs after cardiac arrest. Insights from clinical trials in cardiac arrest may provide direction for future early brain injury research after subarachnoid hemorrhage (SAH). *Methods*. A search of PubMed from 1980 to 2012 and clinicaltrials.gov was conducted to identify published and ongoing randomized clinical trials in aneurysmal SAH and cardiac arrest patients. Only English, adult, human studies with primary or secondary mortality or neurological outcomes were included. *Results*. A total of 142 trials (82 SAH, 60 cardiac arrest) met the review criteria (103 published, 39 ongoing). The majority of both published and ongoing SAH trials focus on delayed secondary insults after SAH (70%), while 100% of cardiac arrest trials tested interventions within the first few hours of ictus. No SAH trials addressing treatment of early brain injury were identified. Twenty-nine percent of SAH and 13% of cardiac arrest trials showed outcome benefit, though there is no overlap mechanistically. *Conclusions*. Clinical trials in SAH assessing acute brain injury are warranted and successful interventions identified by the cardiac arrest literature may be reasonable targets of the study.

## 1. Introduction

For decades, research efforts in subarachnoid hemorrhage (SAH) have focused on vasospasm and delayed ischemic neurological deficits. However, brain injury at the time of aneurysm rupture is a significant predictor of functional outcome. Indeed, poor admission neurological status (Hunt-Hess or World Federation of Neurological Surgeons Score), which reflects acute brain injury, is a larger contributor to death or severe disability than delayed cerebral ischemia [1, 2]. However, the mechanism of early brain injury after aneurysm rupture remains elusive and no current therapies are available. 

One possible mechanism of acute injury was described in a small case series of 6 patients with observed recurrent aneurysm rupture either during transcranial Doppler (TCD) or during craniotomy with open skull but intact dura. The investigators report a spike in intracranial pressure (ICP) that developed over 1 minute and then declined over several minutes. This abrupt increase in ICP approached levels near mean arterial pressure and led to a concomitant drop in cerebral blood flow resulting in circulatory arrest, as documented by TCD [61]. This study examined aneurysm rebleeding and does not provide direct evidence that intracranial circulatory arrest occurs with de novo aneurysm rupture. However, inadequate cerebral blood flow is frequently evidenced clinically by the transient loss of consciousness that occurs at SAH ictus. This mechanism of global transient circulatory arrest has been described in animal models of SAH at the time of initial hemorrhage [62, 63] and mimics the anoxic/hypoxic ischemic mechanism incurred by cardiac arrest. 

In this paper, published and ongoing clinical trials in cardiac arrest are compared to those in aneurysmal SAH to identify overlapping or complementary approaches to treatment as well as new avenues for potential research. 

## 2. Methods 

A search of PubMed was conducted in 11/2012 to identify randomized, controlled trials of aneurysmal SAH and cardiac arrest. Only human studies of adults (≥18 years of age), which tested an intervention published in English between 1980 and 2012, were included. Only trials examining mortality or neurologic outcome as a primary or secondary endpoint were reviewed. Only trials specific to SAH (not trials that included other neurocritical diagnoses or brain injury diagnoses) were included. Cardiac arrest trials included both out-of-hospital and in-hospital arrest and all arrest rhythms were included. Post hoc analyses of preexisting trials were not reviewed. If phase III results of a trial were available, earlier phases of the same trial were not included in analysis unless the patient population or methodology differed substantially. 

A PubMed search of the term “subarachnoid hemorrhage” and “neurologic outcome” with the limits of human, age > 18, English and randomized, controlled trial yielded 23 results. A PubMed search of the term “subarachnoid hemorrhage” and “mortality” with the limits of human, age > 18, English and randomized, controlled trial yielded 78 results. An additional review of articles identified by a broader search of “subarachnoid hemorrhage” with the limits of human, age > 18, English and randomized, controlled trial yielded 244 results. Review of these studies yielded 57 aneurysmal SAH trials that met inclusion criteria and were analyzed. A pubmed search of the terms “cardiac arrest” and “neurologic outcome” with the limits of human, English, age > 18 and randomized, controlled trial yielded 21 results. A PubMed search of the terms “cardiac arrest” and “mortality” with the limits of human, English, age > 18 and randomized, controlled trial yielded 197 results. Review of these studies yielded 46 cardiac arrest trials that met inclusion criteria and were analyzed. 

 Clinicaltrials.gov was searched for ongoing interventional trials in cardiac arrest and aneurysmal subarachnoid hemorrhage. Only ongoing studies that were open and recruiting or preparing to recruit were included. Terminated studies were excluded from review. A search of ongoing studies on clinicaltrials.gov for the term “subarachnoid hemorrhage”, limited to interventional studies of adults ≥18 years old, produced 86 results and a search for the term “cardiac arrest” limited to interventional studies of adults with neurologic outcomes produced 46 results. Of these, 25 ongoing SAH trials and 14 cardiac arrest trials met the criteria for review. 

## 3. Results 

### 3.1. Trials Analyzed

A total of 142 trials (82 SAH, 60 cardiac arrest) met review criteria. Of these, 103 were published in peer-reviewed journals and 39 were ongoing studies. Fifty-seven published randomized, controlled studies were identified in the SAH population and 46 in the cardiac arrest population. These studies are reviewed in detail in Tables 1 and 2. Additionally, 25 ongoing SAH trials and 14 ongoing cardiac trials were reviewed (Tables 3 and 4). 

### 3.2. Interventions Studied

The main hypothetical mechanisms of intervention tested in published SAH trials were related to treating or preventing delayed cerebral ischemia (*N* = 40, 70%), preventing aneurysm rebleeding (*N* = 5, 9%), improving aneurysm repair technique (*N* = 5, 9%), improving fluid balance (*N* = 2, 4%), and others (*N* = 3, 5%). Among ongoing SAH trials, mechanisms of study include treating or preventing delayed cerebral ischemia (*N* = 19, 76%), limiting rebleeding (*N* = 1, 4%), improving aneurysm repair (*N* = 1, 4%), seizure control (*N* = 2, 8%), and other (*N* = 2, 8%). There are no published or ongoing SAH clinical trials that focus on treating acute brain injury after aneurysm rupture. 

 Conversely, the main mechanisms of intervention studied in *published *cardiac arrest trials focused on acute intervention to treat and limit early brain injury. All 46 (100%) published cardiac arrest trials focused on the acute time frame (first few hours) after cardiac arrest. Interventions studied included decreasing cerebral metabolic demand with hypothermia or barbiturate (*N* = 6, 13%), high-quality chest compressions or pressor use to return cerebral blood flow (*N* = 16, 35%), electrolyte/metabolic optimization with calcium, magnesium, sodium bicarbonate or insulin administration (*N* = 12, 26%), neuroprotective drugs including calcium channel blockers (*N* = 5, 11%), thrombolysis to treat the underlying cause of cardiac arrest (*N* = 2, 4%) and other (*N* = 5, 11%). Among *ongoing* cardiac arrest trials, mechanisms of study include decreasing cerebral metabolic demand with hypothermia (*N* = 9, 64%), high-quality chest compressions to return cerebral blood flow (*N* = 2, 14%) electrolyte/metabolic optimization with magnesium (*N* = 1, 7%), neuroprotective drugs (*N* = 1, 7%), and monitoring cerebral oxygenation (*N* = 1, 7%). A detailed list of interventions from published and ongoing studies in both the SAH and cardiac arrest population are listed in Table 5. 

### 3.3. Outcome Measures

The most common neurological outcomes assessed in the SAH trials were delayed cerebral ischemia (*N* = 24, 42%), functional outcome (Glasgow outcome scale, modified Rankin scale or functional outcome scale, *N* = 24, 42%), angiographic or transcranial Doppler vasospasm (*N* = 6, 11%), and death (*N* = 4, 7%). Among cardiac arrest trials, the most often assessed neurological outcomes were the Pittsburgh cerebral performance score (*N* = 18, 40%), Glasgow outcome score or modified Rankin Score (*N* = 4, 9%), Glasgow coma score (*N* = 4, 9%), awakening and command following (*N* = 3, 7%), cognitive or neuropsychological testing (*N* = 1, 2%), “disability” (*N* = 1, 2%), death (*N* = 13, 30%), discharge disposition (*N* = 1, 2%) and others (*N* = 1, 2%). 

### 3.4. Trial Results

Of the clinical trials reviewed for SAH, 30% (17/57) showed that the intervention tested had a statistically significant impact on neurological outcome or mortality. These include studies of nimodipine [4–6, 8, 110], phase II data for nicardipine implants during aneurysm clipping [13], the neuroprotectants edavarone [21] and nizofenone [20], pravastatin [25], early aneurysm surgery [28], endovascular coiling [29–31], cilostazol [41], methylprednisolone [44], erythropoietin [45], and fasudil [57, 58]. Similarly, 30% (17/57) of studies showed a positive impact on delayed cerebral ischemia, infarction, angiographic or TCD vasospasm, though there was incomplete overlap with the above studies that showed outcome benefit. Eight studies found both a significant improvement in delayed cerebral ischemia/vasospasm/infarction and outcome including studies of nimodipine [4, 6, 8], nicardipine implants in the basal cistern [13], edavarone [21], pravastatin [25], fasudil [57], and erythropoietin [45]. Nine studies found a benefit for decreasing delayed cerebral ischemia/vasospasm/infarction but no neurologic outcome benefit including studies of IV nicardipine [10, 11], eicosapentaenoic acid (omega-3 fatty acid) [22], the neuroprotectant NA-1 [23], simvastatin [24], tirilazad [34], intracisternal rTPA [36, 38], and clazosentan [49, 50]. Three studies found improved neurologic outcome despite an insignificant effect on delayed cerebral ischemia/vasospasm/infarction including studies of cilostazol [41], methylprednisolone [44], and fasudil [58]. 

 Among the cardiac arrest trials, 13% (6/46) demonstrated neurologic or mortality benefit. Improved mortality rates were demonstrated with mild therapeutic hypothermia [89], coenzyme Q10 [68], vasopressin plus epinephrine plus methylprednisolone [71], active compression-decompression CPR [93], and hemofiltration [108]. Improved neurological outcome was demonstrated with early mild therapeutic hypothermia for ventricular fibrillation and pulseless ventricular tachycardia arrests [88, 89], and one study of active compression-decompression CPR [93], though a larger study of active compression-decompression was negative [94]. 

### 3.5. Trial Overlap

Though nimodipine has demonstrated mortality and functional outcome benefit in SAH [4–6, 8, 110], it has shown no benefit in cardiac arrest trials [64, 65, 67]. Similarly, intracisternal thrombolysis showed some benefit in reducing delayed cerebral ischemia and infarction after SAH [36, 38], but intravenous tenecteplase showed no long-term benefit and, in fact, increased intracranial hemorrhage after cardiac arrest [69, 70]. Neither magnesium [53, 54, 81–85] nor intensive insulin [59, 87] has proven beneficial after SAH or cardiac arrest. Though hypothermia [88, 89] has been the single most effective treatment for cardiac arrest (the number needed to treat to prevent one death is 7 and the number needed to treat to produce favorable neurological outcome is 6), it has not proven useful in the context of aneurysm surgery after SAH [60]. There is little mechanistic overlap in ongoing randomized, controlled trials of SAH and cardiac arrest patients. 

## 4. Discussion

In this paper, a direct comparison is made between randomized, controlled clinical trials that evaluate mortality or neurologic outcome after SAH and cardiac arrest. Though 28% of SAH studies showed some neurologic outcome benefit in the intervention group, only nimodipine [4–6, 8, 110], fasudil [57, 58], and endovascular coiling [29–31] have been found to consistently improve outcome in multiple, multi-center randomized controlled trials. Smaller studies [8, 41, 58], single center [21, 44], or phase II safety and feasibility studies [13, 25, 45] have shown outcome benefit, but still require larger efficacy trials before integration into standard practice. Among cardiac arrest trials, only mild therapeutic hypothermia has been shown to improve both mortality and neurologic outcome [88, 89]. Little overlap in trial results or mechanisms of study was identified in these different patient populations. 

Methodological differences in the timing, duration, neurological severity, and outcomes studied may explain some of the differences in trial results between SAH and cardiac arrest populations. First, the timing of intervention for SAH and cardiac arrest trials is quite different. With the exception of aneurysm repair and aneurysm rebleeding trials (some of which were carried out in the era of delayed surgical treatment), the vast majority of SAH trials focus on the delayed cerebral ischemia period. Conversely, all cardiac arrest trials are directed at intervening against early brain injury. The difference in time frames studied may explain, in part, the variable results for mild therapeutic hypothermia in each population. Unlike the cardiac arrest trials, which applied hypothermia either prior to ED arrival [88] or within a median of 105 minutes from return of spontaneous circulation (ROSC) [89] for a duration of 12–24 hours, hypothermia was applied in the IHAST trial at a median of two days from SAH onset and only for a brief time (median 5-6 hours) [60]. Second, patient selection may result in variable trial results for hypothermia. For example, hypothermia for cardiac arrest was used for comatose survivors, while relatively neurologically intact patients (WFNS I–III) were studied in the IHAST trial. Finally, outcome measures differ in the cardiac arrest and SAH literature. Many cardiac trials measure 30-day or discharge mortality or neurologic outcome, while SAH trials measure outcomes from 3 months to 1 year. Though the majority of cardiac arrest trials measure neurologic outcome using the Pittsburgh cerebral performance scale, while SAH trials utilize the Glasgow outcome scale or modified Rankin scale, all of these scales are very similar and provide gross estimates of disability. Despite the aforementioned methodological differences, certain interventions, such as magnesium and intensive insulin, have not proven effective in either population. 

Another reason for variable outcome in clinical trials may be due to pathophysiological differences in SAH and cardiac arrest. Though early brain injury in SAH may mechanistically mirror the cascade of injury occurring after cardiac arrest, SAH differs from cardiac arrest in that it is not a monophasic disease. Break down of blood products initiates a distinctive series of delayed clinical events that characteristically can lead to ischemia or infarction between SAH days 3–14. The fact that nimodipine has been so successful in SAH trials, but shown no effect at similar doses in cardiac arrest trials suggests it is acting on a distinct pathway. Indeed, the absolute risk reduction for poor outcome after SAH in a meta-analysis of 16 trials of nimodipine is 5.3% with a number needed to treat for benefit of 19 [111]. No such signal for benefit was seen in cardiac arrest trials [64, 65, 67]. The mechanism of beneficial effect of nimodipine in SAH has been widely debated and may be related to its effect on fibrinolysis [112], spreading cortical depression [113], or excitotoxicity. Though nimodipine improves ischemic neurological deficits by clinical criteria and CT-documented infarction (with a pooled relative risks of 0.66 (95% CI 0.59–0.75) and 0.78 (95% CI 0.70–0.87), resp.) [111], it has little effect on angiographic vasospasm or cerebral blood flow [4, 5]. The corollary to this observation is that interventions that improve angiographic vasospasm, such as clazosentan, do not necessarily improve cerebral infarction or outcome. [49, 50, 114, 115]. While angiographic vasospasm seems to be related to infarction [116], other mechanisms may play a role in neurological deficits, cerebral infarction, and outcome. Such pathophysiological differences may make extrapolation of results from cardiac arrest trials to an SAH population problematic. Indeed, delayed cerebral ischemia (DCI) may blunt the positive effect of hypothermia on early brain injury. Further animal research may better identify mechanistic differences of early brain injury in cardiac arrest and SAH. 

 Despite a second wave of neurological injury in SAH, poor-grade (Hunt Hess 4-5) SAH patients, who are at higher risk for secondary neurological injury, still have comparable, if not better, outcomes compared to cardiac arrest patients who are not cooled. Among Hunt-Hess grade 4-5 patients, the 12-month mortality rate with aggressive treatment is 43%, while 40% had no or slight-moderate disability (mRS 0–3) [117]. By comparison, the 6-month death rate in the control (nonhypothermia) group of the HACA trial was 55%, while good neurologic outcome (defined as Pittsburgh cerebral performance scale 1-2; good outcome or moderate disability) occurred in 26–39% [88, 89]. We have additionally shown that DCI does not predict mortality after SAH with aggressive vasospasm treatment, while early brain injury (measured by Hunt-Hess grade) does [1]. Thus, despite secondary neurologic insults and delayed cerebral ischemia risk, poor-grade SAH patients do at least as well as normothermic cardiac arrest patients, who may face risks to survival and functional outcome related to the underlying cause of the cardiac arrest. Also, the median age of cardiac arrest patients tends to be older than SAH patients, which may also explain why even the sickest SAH patients have relatively good outcomes by comparison. If nihilism can be overcome in the management of poor-grade SAH patients, the early application of mild therapeutic hypothermia may improve outcomes further. 

There are some limitations to this review that should be mentioned. A medical librarian was not used and only MEDLINE/PubMed and clinicaltrials.gov were used to identify literature for review. An Embase search was not performed. Additionally, an exhaustive search for all neurologic outcome based RCTs was not performed, rather only English studies in humans were included. 

In conclusion, while the mechanisms of early brain injury after SAH and cardiac arrest may be similar, the preponderance of SAH clinical trials do not focus on interventions addressing early brain injury. Clinical trials in SAH assessing interventions that have proven successful in the cardiac arrest literature, such as early mild therapeutic hypothermia, are warranted. 

## Figures and Tables

**Table 1:** Randomized controlled trials assessing neurologic outcomes after aneursymal Subarachnoid hemorrhage—completed trials.

Trial name	Study design	Treatment group	Control group	Outcome measure	Results	Reference
Calcium channel blockers—nimodipine						

Cerebral Arterial Spasm—a controlled Trial of Nimodipine in Patients with Subarachnoid Hemorrhage	Randomized, placebo-controlled, double-blind, multicenter prospective study of Hunt Hess grade I-II SAH patients	Nimodipine 0.7 mg/kg PO bolus, then 0.35 mg/kg q 4 × 21 days. Starting within 96 h of SAH (*N* = 58)	Placebo (*N* = 63)	Primary outcome: neurological deficit from arterial spasm and severity of neurologic deficit at 21 days	Nimodipine significantly reduced death or severe deficits from spasm at 21 days (2% versus 13% with placebo, *P* = 0.03)	Allen et al., NEJM 1983 [3]
Nimodipine treatment in poor-grade aneurysm patients. Results of a multicenter double-blind placebo-controlled trial	Randomized, multicenter, double-blind, placebo-controlled trial	Nimodipine 90 mg PO q 4 h × 21 d (*N* = 91)	Placebo (*N* = 97)	Primary outcome: 3-month GOS Secondary outcomes: delayed ischemic deficits, angiographic vasospasm	Better 3-month GOS in treatment group (29% versus 9% of treatment group, *P* < 0.001). Significantly less delayed cerebral ischemia in treatment group, no difference in angiographic vasospasm	Petruk et al., J Neurosurg 1988 [4]
Controlled study of nimodipine in aneurysm patients treated early after subarachnoid hemorrhage	Randomized, double-blind, placebo-controlled trial of all Hunt-Hess grades within 96 hours of SAH	Nimodipine 60 mg q 4 h PO × 21 days + Nimodipine 200 mcg IV intraoperatively into basal cistern (*N* = 38)	Placebo (*N* = 37)	Primary outcome: mortality, cerebral blood flow measured by Xenon CT Secondary outcomes: 3-month intellectual or neurological deficit	Mortality was lower in the nimodipine group (4% versus 24% with placebo, *P* < 0.05). Nimodipine did not significantly increase cerebral blood flow	Mee et al., Neurosurgery 1988 [5]
Effect of oral nimodipine on cerebral infarction and outcome after subarachnoid hemorrhage: British aneurysm nimodipine trial	Randomized, double-blind, placebo-controlled, multicenter trial within 96 h of SAH	Nimodipine 60 mg q 4 PO × 21 d (*N* = 276)	Placebo (*N* = 278)	Primary outcome: 3-month cerebral infarction Secondary outcome: 3-month GOS	Significantly less cerebral infarction in the nimodipine group (22% compared to 33% in placebo, *P* = 0.014). Poor GOS outcomes significantly reduced in nimodipine group at 3-months	Pickard et al., BMJ 1989 [6]
Early aneurysm surgery and preventive Therapy with intravenously administered nimodipine: A multicenter, double-blind, dose-comparison study	Randomized, double-blind, dose-comparison, multicenter study	Nimodipine 2 mg/h IV for 9–15 days (*N* = 101)	Nimodipine 3 mg/h IV for 9–15 days (*N* = 103)	Primary outcome: delayed neurological deficits, adverse drug reactions	No difference in delayed neurological deficits between the two groups	Gilsbach et al., Neurosurgery 1990 [7]
Long-term effects of nimodipine on cerebral infarcts and outcome after aneurysmal subarachnoid hemorrhage and surgery	Randomized, double-blind, placebo-controlled of Hunt-Hess I–III SAH patients	Nimodipine IV 0.5 mcg/kg/min × 7–10 days followed by 60 mg q 4 h PO × 21 days total (*N* = 104)	Placebo (*N* = 109)	Primary outcome: delayed ischemic deterioration and CT infarcts Secondary outcomes: GOS at 1–3 years	Significantly fewer deaths caused by delayed cerebral ischemia in nimodipine group (*P* = 0.01) and fewer cerebral infarcts on CT (*P* = 0.05). No differences in 1–3 year GOS or CT scan	Ohman et al., J Neurosurg 1991 [8]
A randomized outcome study of enteral versus intravenous nimodipine in 171 patients after acute aneurysmal subarachnoid hemorrhage	Randomized, single-center study	Nimodipine 2 mg/h IV × 10 days then changed to PO × 6 d (*N* = 87)	Nimodipine 60 mg PO q 4 × 16 days (*N* = 84)	Primary outcome: delayed ischemic neurological deficit Secondary outcomes: 12 month GOS, mRS, Karnofsky, MRI infarcts, HRQoL	No difference in delayed ischemic neurological deficits (20% in enteral versus 16% in IV group, *P* = 0.61), no difference in 12-month clinical outcomes	Soppi et al., World Neurosurgery 2012 [9]

Calcium channel blockers—nicardipine						

A randomized controlled trial of high-dose intravenous nicardipine in aneurysmal subarachnoid hemorrhage. A report of the cooperative aneurysm study	Randomized, double-blind, placebo-controlled, multicenter study	Nicardipine IV 0.15 mg/kg/h (*N* = 449)	Placebo (*N* = 457)	Primary outcome: 3-month GOS Secondary outcomes: angiographic vasospasm, TCD vasospasm, mortality, disability from vasospasm, symptomatic vasospasm, CT infarction, NIHSS	No difference in 3-month GOS. Less symptomatic vasospasm in treatment group (32% versus 46% in placebo group, *P < *0.001) and less angiographic vasospasm in treatment group (33% versus 51% of placebo, *P < *0.01) and less TCD vasospasm (23% versus 49% of placebo, *P < *0.001)	Haley et al., J Neurosurg 1993 [10, 11]
A randomized trial of two doses of nicardipine in aneurysmal subarachnoid hemorrhage. A report of the cooperative aneurysm study	Randomized, double-blind, multicenter study	Nicardipine IV 0.15 mg/kg/h × 14 days (*N = *184)	Nicardipine IV 0.075 mg/kg/h × 14 days (*N = *181)	Primary outcome: symptomatic vasospasm, adverse drug events Secondary outcomes: 3-month GOS and NIHSS, mortality, disability due to vasospasm, CT infarction	No difference in symptomatic vasospasm or 3-month outcome. More adverse effects in high-dose nicardipine group	Haley et al., J Neurosurg 1994 [12]
Effect of nicardipine prolonged-release implants on cerebral vasospasm and clinical outcome after severe aneurysmal subarachnoid hemorrhage. A prospective, randomized, double-blind phase IIa Study	Randomized, prospective double-blind phase IIa study in clipped SAH patients	Nicardipine prolonged-release implants (10 × 4 mg prolonged release rod shaped polymers) placed in basal cisterns (*N = *16)	Control-basal cisterns opened and washed out (*N = *16)	Primary outcome: angiographic vasospasm Secondary outcome: delayed ischemic lesion on HCT, 1-year mRS and NIHSS	Angiographic vasospasm significantly reduced in treatment group (7% versus 73% in controls, *P < *0.05). No significant difference in CT infarct. Decreased mortality in treatment group (6% versus 38% in control group, *P = *0.042) and better 1-year mRS and NIHSS (*P = *0.0001)	Barth et al., Stroke 2007 [13]

Antifibrinolytics						

Antifibrinolysis with tranexamic acid in aneurysmal subarachnoid hemorrhage: A consecutive controlled clinical trial	Randomized, placebo-controlled, study	Tranexamic acid 1 g q 4 h IV × 1 week then 1 g q 6 h IV × 1 week then 1.5 g q 6 h PO × 1 week (*N = *30)	Placebo (*N = *29)	Primary outcome: recurrent hemorrhage diagnosed by LP, HCT, echoencephalogram or autopsy. Secondary outcome: angiographic vasospasm, delayed cerebral ischemia, death	Tranexamic acid protected against rebleeding during the first 2 weeks of treatment but also resulted in cerebral ischemic complications	Fodstad et al., Neurosurgery 1981 [14]
Comparative clinical trial of epsilon amino-caproic acid and tranexamic acid in the prevention of early recurrence of subarachnoid hemorrhage	Randomized trial	Epsilon amino-caproic acid 6 g q 6 h IV continued until surgery or discharge (*N = *90)	Tranexamic acid 1 g q 6 h IV continued until surgery or discharge (*N = *61)	Primary outcome: recurrent hemorrhage diagnosed clinically by HCT, LP, or autopsy Secondary outcome: delayed ischemic deficit diagnosed by clinical deterioration, angiographic vasospasm, and infarct on HCT	Rebleeding occurred in 8% of aminocaproic-acid-treated patients and 10% of tranexamic acid treated patients. Delayed ischemic deficits occurred in 7% of aminocaproic acid patients and 5% of tranexamic acid patients. Mortality was 11% in each group. *P = *NS for all outcomes	Chowdhary and Sayed, JNNP 1981 [15]
Antifibrinolytic treatment in subarachnoid hemorrhage	Randomized, double-blind, placebo-controlled, multicenter study	Tranexamic acid 1 g q 4 h IV × 1 week then 1 g q 6 h IV × 3 weeks (*N* = 241)	Placebo (*N = *238)	Primary outcome: 3-month GOS Secondary outcome: neurological deterioration, rebleeding, infarction, hydrocephalus, edema, epilepsy	Rebleeding reduced from 24% in control group to 9% in treatment group (*P < *0.001), but with concurrent increase in ischemic complications (24% in treatment group versus 15% in placebo, *P < *0.01). No difference in 3 month GOS	Vermeulen et al., NEJM 1984 [16]
Antifibrinolytic treatment in subarachnoid hemorrhage: a randomized placebo-controlled trial (STAR)	Prospective, double-blind, placebo-controlled, multicenter, randomized trial within 96 hours of SAH onset in whom aneurysm repair was delayed beyond 48 hours	Tranexamic acid 1 g IV q 4 h × 1 week then 1.5 g PO q 6 h × 2 weeks (*N = *229)	Placebo (*N = *233)	Primary outcome: 3-month GOS Secondary outcomes: rebleeding, delayed cerebral ischemia, hydrocephalus, postoperative ischemia	No difference in 3-month GOS. Significant decrease in rebleeding from 33% in placebo group to 19% in treatment group. No difference in delayed cerebral ischemia, hydrocephalus, or postoperative ischemia	Roos, Neurology 2000 [17]
Immediate administration of tranexamic acid and reduced incidence of early rebleeding after aneurysmal subarachnoid hemorrhage: a prospective randomized study	Randomized, placebo-controlled trial	Tranexamic acid 1 g IV bolus, then 1 g IV q 6 hours until aneurysm repair or 72 hours post ictus. (*N = *254)	Placebo (*N = *251)	Primary outcome: Rebleeding by HCT Secondary outcome: 6 -month GOS, clinical vasospasm/delayed ischemic neurological deficit, TCD spasm	Treatment group had reduced rebleeding rate of 2.4% compared to 10.8% in the placebo group (*P < *0.01). More favorable outcome in the treatment group (74.8% compared to 70.5% in the control group, *P = *NS). No increased risk of ischemia	Hillman et al., J Neurosurg 2002 [18]

Neuroprotectives drugs						

A double-blind clinical evaluation of the effect of nizofenone on delayed ischemic neurological deficits following aneurysmal rupture	Randomized, placebo controlled trail	Nizofenone for 5–10 days (*N = *42)	Placebo (*N = *48)	Primary outcome: delayed ischemic neurological deficits with angiographically confirmed vasospasm. Secondary outcomes: one-month disability index, motor, and speech function	No difference in delayed ischemic events between treatment groups. Among patients with vasospasm, those who received nizofenone had better one-month functional outcomes (*P < *0.05)	Saito et al., Neurol Res 1983 [19]
Nizofenone administration in the acute stage following subarachnoid hemorrhage. Results of a multicenter controlled double-blind clinical study	Randomized, double-blind, placebo-controlled, multicenter study of Hunt Hess grade I–IV	Nizofenone 5 mg × 2 weeks (*N = *102)	Placebo (*N = *106)	Primary outcome: neurological exam at 1-month and discharge	Significantly improved one-month or discharge functional outcome in treatment group compared to placebo (*P < *0.05). No difference in mortality	Ohta et al., J Neurosurg 1986 [20]
Effect of a free radical scavenger, edaravone, in the treatment of patients with aneurysmal subarachnoid hemorrhage	Randomized, controlled, single-center study	Edaravone 30 mg IV BID × 14 days (*N = *49)	Control (usual treatment) (*N = *42)	Primary outcome: delayed ischemic neurological deficits Secondary outcomes: cerebral infarction due to vasospasm, 3-month GOS	No difference in delayed ischemic neurological deficits between treatment and control groups. Less cerebral infarction in treatment group (0% versus 66%, *P = *0.028). Poor outcome caused by vasospasm 0% in treatment group and 71% in control group (*P = *0.046)	Munakata et al., Neurosurgery 2009 [21]
Eicosapentaenoic Acid Cerebral Vasospasm Therapy Study (EVAS)	Randomized, controlled, open label, multicenter, efficacy study of surgically clipped SAH patients	Eicosapentaenoic acid (omega 3 fatty acid) 900 mg TID × 30 days (*N = *81)	Control (usual treatment) (*N = *81)	Primary outcome: symptomatic vasospasm or infarct on HCT Secondary outcome: 1-month GOS	Symptomatic vasospasm occurred significantly less in the treatment group (15% versus 30% in controls, *P = *0.022) as did infarction from vasospasm (7% versus 21% in controls, *P = *0.012)	Yoneda et al., World Neurosurg 2012 [22]
Safety and efficacy of NA-1 in patients with iatrogenic stroke after endovascular aneurysm repair (ENACT): a phase 2, randomized, double-blind, placebo-controlled trial	Randomized, double-blind, placebo-controlled study of ruptured (WFNS 1–3) and unruptured aneurysms undergoing endovascular repair	NA-1 2.6 mg/kg infusion over 10 minutes (*N = *92)	Placebo (*N = *93)	Primary outcome: safety, number and volume of ischemic strokes on MRI DWI and FLAIR 12–96 hours after infusion Secondary outcome: 30-day mRS, NIHSS, neurocognitive outcome	No difference in MRI lesion volume, but fewer ischemic lesions in NA-1 group compared to placebo (*P = *0.012). In the SAH subgroup (20% of cohort) their MRI number and ischemic volume was significantly less in the treatment group. No difference in 30 day NIHSS or mRS between groups	Hill et al., Lancet Neurol 2012 [23]

Statins						

Simvastatin reduces vasospasm After aneurysmal subarachnoid hemorrhage: results of a pilot randomized clinical trial	Randomized, placebo-controlled pilot trial	Simvastatin 80 mg qd for 14 days (*N = *19)	Placebo (*N = *20)	Primary outcome: delayed ischemic neurological deficit confirmed by TCD or angiography. Secondary outcomes: liver transaminases, CK, von Willebrand factor, S100	Vasospasm occurred in 26% of treatment group compared to 60% of placebo group (*P < *0.05). No differences in transaminitis or myositis. VWF and S100 were significantly lower in the treatment group (*P < *0.05)	Lynch et al., Stroke 2005 [24]
Effects of acute treatment with pravastatin on cerebral vasospasm, autoregulation, and delayed Ischemic deficits after aneurysmal subarachnoid hemorrhage. A phase II randomized placebo-controlled trial	Randomized placebo-controlled, phase II Trial	Pravastatin 40 mg PO qd × 14 d (*N = *40)	Placebo (*N = *40)	Primary outcome: incidence, severity, and duration of vasospasm on TCD, duration of impaired autoregulation measured by transient hyperemic response on TCD Secondary outcome: vasospasm-related delayed ischemic deficits, disability at discharge	TCD vasospasm and severe vasospasm were reduced in the treatment group (*P = *0.006 and *P = *0.044, resp.). Duration of impaired autoregulation shortened in treatment group (*P < *0.01). Vasospasm-related delayed ischemic deficits was reduced (*P < *0.001) and mortality was reduced (*P = *0.037)	Tseng et al., Stroke 2005 [25]
A randomized, double-blind, placebo-controlled pilot study of simvastatin in aneurysmal subarachnoid hemorrhage	Randomized, double-blind, placebo-controlled pilot study	Simvastatin 80 mg qd in statin naïve Fisher 3 SAH until discharge or 21 days (*N = *19)	Placebo (*N = *20)	Primary outcome: death and drug morbidity (elevated CK, transaminases) Secondary outcomes: TCD, angiographic or clinical vasospasm, vasospasm-related infarcts, clinical outcomes at discharge, cardiac, and infectious morbidities	Mortality in 0% treatment group and 15% placebo group. Angiographically confirmed vasospasm in 26% treatment group and 25% placebo group. Vasospasm infarcts in 11% treatment group and 25% placebo group. All differences *P = *NS	Chou et al., Stroke 2008 [26]
Biological effects of simvastatin in patients with aneurysmal subarachnoid hemorrhage: a double-blind, placebo-controlled randomized trial	Double-blind, placebo-controlled randomized trial	Simvastatin 80 mg PO × 15 days (*N = *16)	Placebo (*N = *16)	Primary outcome: effect of simvastatin on laboratory parameters of endothelial function, fibrinolysis, coagulation, inflammation and cholesterol Secondary outcomes: TCD vasospasm, clinical signs of DCI, 3- and 6-month GOS	Simvastatin group had significantly lower total cholesterol and LDL, but no differences in coagulation, fibrinolysis, endothelium function, or inflammation. No differences in TCD vasospasm, clinical DCI, or poor outcome	Vergouwen et al., J Cereb Blood Flow Metab 2009 [27]

Aneurysm repair						

Timing of operation for ruptured supratentorial aneurysms: a prospective randomized study	Randomized, prospective study of Hunt Hess grade I–III SAH patients	Acute surgery (day 0–3 after SAH) (*N = *71)	Intermediate surgery (day 4–7 after SAH) (*N = *70) Late surgery (day 8 or later after SAH) (*N = *70)	Primary outcome: 3-month dead, dependent or independent. Secondary outcomes: Neurological deficit from direct effect of initial bleed, complication of surgery, confirmed rebleeding, delayed ischemic deterioration, hydrocephalus, extracranial complications	Acute surgery patients were more often independent at 3-months (92% versus 79% in intermediate timing and 80% in the late timing group, *P < *0.01). Mortality was 6% in the early surgery group versus 13% in the late surgery group (*P = *NS)	Ohman and Heiskanen, J Neurosurg 1989 [28]
International subarachnoid aneurysm trial (ISAT) of neurosurgical clipping versus endovascular coiling in 2143 patients with ruptured intracranial aneurysms: a randomized trial	Randomized, unblinded trial of SAH patients with an aneurysm judged technically suitable for either clipping or coiling and clinical equipoise	Surgical clipping (*N = *1070)	Endovascular treatment by detachable platinum coils (*N = *1073)	Primary outcome: 1-year mRS 3–6 versus 1-2 Secondary outcomes: rebleeding, quality of life at 1 year (euroQol), frequency of epilepsy, cost effectiveness, neuropsychological outcomes	Dependent or dead at 1 year: 23.7% endovascular versus 30.6% clipping (*P = *0.0019).	Molyneux et al., ISAT Collaborative Group, Lancet 2002 [29]
International subarachnoid aneurysm trial (ISAT) of neurosurgical clipping versus endovascular coiling in 2143 patients with ruptured intracranial aneurysms: a randomized comparison of effects on survival, dependency, seizures, rebleeding, subgroups, and aneurysm occlusion	Randomized, unblinded trial of SAH patients with an aneurysm judged technically suitable for either clipping or coiling and clinical equipoise	Surgical clipping (*N = *1070)	Endovascular treatment by detachable platinum coils (*N = *1073)	Primary outcome: 1-year mRS 3–6 versus 1-2 Secondary outcomes: rebleeding, quality of life at 1-year (Euroqol), frequency of epilepsy, cost effectiveness, neuropsychological outcomes	Dead or dependent at 1-year: 23.5% of endovascular group versus 30.9% of clipping group. ARR 7.4%. Early survival advantage of coiling maintained up to 7 years (*P = *0.03). Lower risk of epilepsy in coiled group but higher late rebleeding risk in coiled group	Molyneux et al., Lancet 2005 [30]
The barrow ruptured aneurysm trial	Randomized, open-label, prospective, single-center study	Surgical clipping (*N = *238)	Endovascular coiling (*N = *233)	Primary outcome: 1-year mRS > 2	Poor outcome in 33.7% of clipped and 23.2% of coiled patients (*P = *0.02)	McDougall et al., J Neurosurg 2012 [31]

Lipid peroxidation inhibitor						

Randomized, double-blind, vehicle-controlled trial of tirilazad mesylate in patients with aneurysmal subarachnoid hemorrhage: a cooperative study in Europe, Australia, and New Zealand	Double-blind, randomized, vehicle-controlled study in men and women with aneurysmal SAH	Tirilazad 0.6 mg/kg/ (*N = *257) Tirilazad 2 mg/kg/d (*N = *249) Tirilazad 6 mg/kg/d (*N = *256) up to 11 days	Placebo containing citrate vehicle (*N = *253)	Primary outcome: Symptomatic vasospasm Secondary outcome: 3-month GOS, NIHSS, infarct volume on head CT	The subgroup 6 mg/kg treatment arm had reduced mortality (*P = *NS) and better 3-month GOS (*P = *NS) compared to placebo. Less symptomatic vasospasm in 6 mg/kg group, but not significant. Men showed more benefit than women. No significant improvement with lower dosing groups	Kassell et al., J Neurosurg 1996 [32]
A randomized, double-blind, vehicle-controlled trial of tirilazad mesylate in patients with aneurysmal subarachnoid hemorrhage: a cooperative study in North America	Double-blind, randomized, vehicle-controlled study in men and women with aneursymal SAH	Tirilazad 2 mg/kg/d (*N = *298) Tirilazad 6 mg/kg/d (*N = *299) up to 11 days	Placebo containing citrate vehicle (*N = *300)	Primary outcome: mortality at 76 days Secondary outcome: 3-month GOS and NIHSS, infarct volume on head CT symptomatic vasospasm, incidence, and severity of angiographic vasospasm	No difference in mortality, favorable GOS outcome, or employment between groups. No differences in symptomatic or angiographic vasospasm	Haley et al., J Neurosurg 1997 [33]
Double-blind, randomized, vehicle-controlled study of high-dose tirilazad mesylate in women with aneurysmal subarachnoid hemorrhage. Part I a cooperative study in Europe, Australia, New Zealand, and South Africa	Double-blind, randomized, vehicle-controlled study in women with aneurysmal SAH	Tirilazad mesylate 15 mg/kg/d IV hours for 11 days (*N = *405)	Placebo containing citrate vehicle (*N = *414)	Primary outcome: 91-day mortality Secondary outcome: 3-month GOS, clinical vasospasm, use of hypervolemic hypertensive therapy, neurological worsening from vasospasm, cerebral infarction, use of angioplasty, safety endpoints	Mortality rates and 3-month GOS not different between groups. Lower symptomatic vasospasm in tirilazad group (24.8% versus 33.7% in placebo group, *P = *0.005). Cerebral infarction 8% in treatment group versus 13% in placebo group (*P < *0.04)	Lanzino et al., J Neurosurg 1999 [34]
Double-blind, randomized, vehicle-controlled study of high-dose tirilazad mesylate in women with aneursymal subarachnoid hemorrhage. Part II a cooperative study in North America	Double-blind, randomized, vehicle-controlled study in women with aneurysmal SAH	Tirilazad mesylate 15 mg/kg/d IV up to 11 days (*N = *410)	Placebo containing citrate vehicle (*N = *413)	Primary outcome: mortality at 91 days in WFNS grade IV-V patients Secondary outcomes: 3 month GOS or clinical vasospasm 1–14 days from dosing, use of hypervolemic hypertensive therapy, neurological worsening from vasospasm, cerebral infarction, use of angioplasty, safety endpoints	No differences in mortality when analyzing the entire population. No difference in GOS, symptomatic vasospasm, vasospasm severity. In WFNS grades IV-V, lower mortality in treatment group (24.6% versus 43.4% in placebo, *P = *0.016). In WFNS I–III improved GOS in placebo group (83.3% versus 76.7% in treatment group, *P = *0.04)	Lanzino and Kassell, J Neurosurg 1999 [35]

Thrombolytics						

Prevention of delayed ischemic deficits after aneurysmal subarachnoid hemorrhage by intrathecal bolus injection of tissue plasminogen activator (rTPA)	Prospective, controlled trial of Fisher III clipped SAH patients	rTPA 10 mg IV intracisternal immediately following aneurysm clipping ± 5–10 mg IV TPA intraventricularly in patients with IVH (*N = *52)	No TPA instillation (*N = *68)	Primary outcome: clinical delayed ischemic deficits attributed to vasospasm Secondary outcome: 3-month GOS	Significantly less transient and permanent delayed ischemic deficits and better GOS in rTPA group	Seifert et al., Acta Neurochir 1994 [36]
A randomized trial of intraoperative, intracisternal tissue plasminogen activator for the prevention of vasospasm	Randomized, double-blinded, placebo-controlled, multicenter study	rTPA 10 mg intracisternal at the time of aneurysm clipping (*N = *51)	Placebo vehicle (*N = *49)	Primary outcome: angiographic vasospasm Secondary outcome: mortality, 3-month GOS, symptomatic vasospasm, clot clearance on CT, TCD velocities, use of HHH on angioplasty to treat vasospasm	No difference in angiographic vasospasm, vasospasm treatment, TCD velocities, mortality, or 3-month GOS	Findlay, Neurosurgery 1995 [37]
Efficacy of low-dose tissue-plasminogen activator intracisternal administration for the prevention of cerebral vasospasm after subarachnoid hemorrhage	Randomized, controlled trial	Intermittent Tisokinase 960,000 IU via cisternal drain (*N = *20) Continuous infusion Tisokinase 1920 IU/h × 48 h via cisternal drain (*N = *20)	Control (standard treatment) (*N = *20)	Primary outcome: clearance of subarachnoid clots by HCT Secondary outcome: delayed cerebral ischemia, 3-month mRS and GOS	Subarachnoid clot by HCT and delayed cerebral ischemia were significantly less in the treatment groups compared to control (*P < *0.05). The intermittently treated group had better neurological outcomes than the control group (*P < *0.05)	Yamamoto et al., World Neurosurgery 2010 [38]

Anti-platelets						

Dipyridamole and postoperative ischemic deficits in aneurysmal subarachnoid hemorrhage	Randomized, placebo-controlled, single-blind controlled trial	Dipyridamole 100 mg PO qd or 10 mg/day IV × 3-months (*N = *336)	Placebo (*N = *314)	Primary outcome: 3-month GOS Secondary outcome: neurological deterioration following aneurysm repair	No differences in 3-month GOS or delayed neurological deterioration	Shaw et al., J Neurosurg 1985 [39]
Randomized controlled trial of acetylsalicylic Acid in aneurysmal subarachnoid hemorrhage: the MASH study	Randomized controlled pilot study; factorial design (magnesium versus placebo and ASA versus placebo, separated a priori)	Aspirin 100 mg PR qd × 14 days within 12 hours of aneurysm occlusion. (*N = *87)	Placebo (*N = *74)	Primary outcome: delayed ischemic neurological deficits within 3-months of SAH consisting of HCT infarcts plus clinical decline Secondary outcome: new CT infarcts of any cause, postop hemorrhage, mRS ≥ 4, mRS ≥ 1	No difference in delayed ischemic events, CT infarction, or 3-month outcomes	Van den Bergh, Stroke 2006 [40]
Cilostazol improves outcome after subarachnoid hemorrhage: a preliminary report	Randomized, single-blind, prospective, multicenter study	Cilostazol 100 mg PO BID (*N = *49)	Control (usual care) (*N = *51)	Primary outcome: symptomatic vasospasm and cerebral infarction, discharge mRS	No difference in symptomatic vasospasm or cerebral infarction. mRS at discharge better in treatment group (1.5 versus 2.6 in controls, *P = *0.041)	Suzuki et al., Cerebrovasc Dis 2011 [41]

Steroids						

Effect of fludrocortisone acetate in patients with subarachnoid hemorrhage	Randomized, placebo controlled, multicenter trial	Fludrocortisone 400 mcg/day BID × 12 days PO or IV (*N = *46)	Placebo (*N = *45)	Primary outcome: plasma volume change, fluid balance, sodium balance Secondary outcome: delayed cerebral ischemia within 28 days and 28-day GOS	Treatment reduced negative sodium balance (*P = *0.014) but did not affect plasma volume. No significant difference in cerebral ischemia (22% versus 31% in controls, *P = *0.349). Similar outcome in each group	Hasan et al., Stroke 1989 [42]
A randomized controlled trial of hydrocortisone against hyponatremia in patients with aneurysmal subarachnoid hemorrhage	Randomized, placebo-controlled study	Hydrocortisone 300 mg q 6 h × 10 d then taper over 4 d (*N = *35)	Placebo (*N = *36)	Primary outcome: hyponatremia < 140 mmol/L Secondary outcome: 30-day mRS, symptomatic vasospasm	Less sodium excretion and urine volume in treatment group (*P = *0.04). No significant differences in vasospasm or mRS	Katayama et al., Stroke 2007 [43]
Randomized, double-blind, placebo-controlled, pilot trial of high-dose methylprednisolone in aneurysmal subarachnoid hemorrrhage	Randomized, double-blind, placebo-controlled, single center study	Methylprednisolone 16 mg/kg IV qd × 3 days (*N = *49)	Placebo (*N = *46)	Primary outcome: symptomatic vasospasm and infarct on HCT Secondary Outcomes: 1 year GOS, functional outcome scale, and severity of delayed ischemic deficits	No significant difference in symptomatic vasospasm or infarct on HCT. No difference in 1-year GOS or delayed ischemic deficits at 3-months. Poor outcome by functional outcome scale was reduced in treatment group (*P = *0.02)	Gomis et al., J Neurosurg 2010 [44]

Transfusion/erythropoietin/albumin						

Acute systemic erythropoietin therapy to reduce delayed ischemic deficits following aneurysmal subarachnoid hemorrhage: a phase II randomized, double-blind, placebo-controlled trial	Phase II randomized, double-blind, placebo-controlled trial	Erythropoietin IV (30,000 u) every 48 h for a total of 90,000 U (*N = *40)	Placebo (*N = *40)	Primary outcome: incidence, duration and severity of TCD vasospasm; duration of impaired autoregulation by TCD Secondary outcome: incidence of delayed ischemic deficits, mRS, GOS, and NIHSS at discharge and 6-months	No differences in incidence of TCD vasospasm or adverse events. Treatment group had less severe TCD vasospasm (*P = *−0.037), reduced delayed ischemic deficits/delayed cerebral infarcts (*P = *0.001), and shortened duration of impaired autoregulation (*P < *0.001) and more favorable discharge outcome (*P = *0.039)	Tseng et al., J Neurosurg 2009 [45]
Prospective, randomized trial of higher goal hemoglobin after SAH	Prospective, randomized pilot safety, and feasibility study	Packed RBC transfusion to goal Hgb 11.5 g/dL (*N = *21)	Packed RBC transfusion to goal Hgb 10 g/dL (*N = *23)	Primary outcomes: days of core temp > 100.4 F, ventilator-free days, hemoglobin level Secondary outcomes: NIHSS, mRS, and MRI at 14 days, mRS at 28 days and 3-months	Higher target Hgb resulted in more transfusions. No difference in safety endpoints. Number of MRI infarcts, NIHSS, and mRS similar between both groups at all timepoints	Naidech et al., Neurocrit Care 2010 [46]
The Albumin in Subarachnoid Hemorrhage multicenter pilot clinical trial: safety and neurologic outcomes (ALISAH)	Open label, dose escalation study	Albumin in 3 tier doses: 0.625 g/kg/d (*N = *20), 1.25 g/kg/d (*N = *20), 1.875 g/kg/d (*N = *7) × 7 days (*N = *47 total)	NA	Primary outcomes: severe to life threatening heart failure, anaphylaxis Secondary outcomes: functional outcome at 3-months	Doses up to 1.25 g/kg/d × 7 days tolerated without dose-limiting complications. Trend toward better outcomes in 1.25 g/kg/d dose compared to 0.625 g/kg/d	Suarez et al., Stroke 2012 [47]

Vasodilators—CRGP and endothelin receptor antagonist						

Effect of calcitonin-gene-related peptide in patients with delayed postoperative cerebral ischemia after aneurysmal subarachnoid hemorrhage	Randomized, single-blind, controlled, multicenter study	Calcitonin-related gene peptide (0.6mcg/min) × 10 days (*N = *62)	Standard medical therapy (*N = *55)	Primary outcome: 3-month GOS	No difference in 3-month GOS. Hypotension common in treatment group.	Bell, European CGRP in subarachnoid Hemorrhage study group, Lancet 1992 [48]
Clazosentan, an endothelin receptor antagonist, in patients with aneurysmal SAh undergoing surgical clipping: a randomized, double-blind, placebo-controlled phase 3 trial (CONSCIOUS 2)	Phase 3 randomized placebo-controlled double-blinded	Clazosentan (5 mg/h IV up to 14 days) (*N = *748)	Placebo (*N = *389)	Primary outcomes: cerebral vasospasm-related morbidity (DCI/DIND/vasospasm therapy), all-cause mortality at 6 weeks Secondary outcomes: 12-week GOSE	No effect on primary endpoint (21% in clazosentan group and 25% in placebo group *P = *NS). Poor outcome (GOSE) in 29% clazosentan and 25% placebo group	MacDonald et al., Lancet Neuol 2011 [49]
Randomized trial of clazosentan in patients with aneurysmal subarachnoid hemorrhage undergoing endovascular coiling (CONSCIOUS 3)	Phase 3 randomized placebo-controlled double-blinded; terminated early for futility (planned *N = *1500)	Clazosentan (5 or 15 mg/h IV up to 14 days) (*N = *194)	Placebo (*N = *189)	Primary outcomes: cerebral vasospasm related morbidity (DCI/DIND/vasospasm therapy), all-cause mortality at 6 weeks Secondary outcomes: 12-week GOSE	Clazosentan 15 mg/h significantly reduced vasospasm-related morbidity/all-cause mortality at 6 weeks but did not improve long-term outcome Primary outcome: 24% in clazosentan 5 mg/h and 27% in placebo group *P = *NS and 15% in clazosentan 15 mg/h *P = *0.007 Poor outcome in 25% of clazosentan 5 mg/h, 28% clazosentan 15 mg/h, and 24% of placebo group *P = *NS.	MacDonald et al., Stroke 2012 [50]

Hypertensive, hypervolemic therapy (prophylactic)						

Effect of hypervolemic therapy on cerebral blood flow after subarachnoid hemorrhage: A randomized controlled trial	Randomized, controlled, single-center study	High-volume management (with colloid and crystalloid) to target PADP ≥ 14 mmHg or CVP ≥ 8 mmHg (*N = *41)	Normal volume management (with colloid and crystalloid) to target PADP ≥ 7 mmHg or CVP ≥ 5 mmHg (*N = *41)	Primary outcome: CBF by Xenon CT and blood volume by tagged RBC Secondary outcomes: symptomatic vasospasm, medical complications, GOS and 3, 6, and 12 months	High-volume management patients received significantly more fluid but there was no effect on net fluid balance or blood volume. No difference in CBF or vasospasm	Lennihan et al., Stroke 2000 [51]
Prophylactic hyperdynamic postoperative fluid therapy after aneurysmal subarachnoid hemorrhage: a clinical, prospective, randomized, controlled trial	Randomized, controlled Prospective, trial of Hunt Hess I–III patients	Hypertensive (MAP 20 mmHg greater than pre-op), hypervolemic (CVP 8–12 mmHg) and hemodilutional (Hct 30–35%) therapy (*N = *16)	Normovolemic crystalloid fluid therapy until day 12 (*N = *16)	Primary outcome: TCD vasospasm, CBF by SPECT on day 12 Secondary outcomes: 1 year GOS, neuropsych outcomes, and SPECT	No differences in TCD vasospasm or SPECT CBF. No difference in 1-year GOS, SPECT, or neuropsych outcomes	Egge et al., Neurosurgery 2001 [52]

Magnesium						

Intravenous magnesium sulfate for aneurysmal subarachnoid hemorrhage (IMASH): a randomized double-blinded, placebo-controlled, multicenter phase III trial	Randomized double-blinded, placebo-controlled, multicenter phase III trial.	MgSO_4_ IV infusion to 2x baseline value (20 mmol over 30 minutes then continuous infusion of 80 mmol/d × 14 days; maximum allowed serum Mg of 2.5 mmol/L (*N = *169)	Equivalent volume of normal saline infusion. Occasional changes in infusion rates to maintain blinding. (*N = *158)	Primary outcome: 6-month GOSE 5–8 Secondary outcome: clinical vasospasm during initial 2 weeks, 6-month mRS, Barthel Index, and Short Form 36	Favorable 6-month GOSE (5–8) 64% of Mg group and 63% placebo (*P = *NS) No difference in mRS, Barthel, Short Form 36, or clinical vasospasm. No subgroup differences	Wong et al., Stroke 2010 [53]
Magnesium for aneurysmal subarachnoid hemorrhage (MASH-2): a randomized placebo-controlled trial	Randomized, double-blind, placebo controlled, multicenter, phase III trial	MgSO_4_ IV 64 mmol/day (*N = *606)	Placebo (*N = *507)	Primary outcome: 3-month mRS 4–6	No difference in poor outcome in the MgSO_4_ group (26.2% versus 25.3% in placebo group)	Mees et al., Lancet 2012 [54]

Adrenergic blockade						

Beneficial effects of adrenergic blockade in patients with subarachnoid hemorrhage	Randomized controlled trial	Phentolamine 20 mg q 3 h + propranolol 80 mg q 8 × 3 weeks (*N = *68)	Placebo (*N = *66)	Primary outcome: neurological deficit at 28 days	Trend toward less neurological deficit in the treated group (*P = *0.053)	Walter et al., BMJ 1982 [55]

Endovascular therapy						

Effect of prophylactic transluminal balloon angioplasty on cerebral vasospasm and outcome in patients with Fisher grade III subarachnoid hemorrhage: results of a phase II multicenter, randomized clinical trial	Unblinded, randomized phase II trial of Fisher III and Fisher III + IV SAH patients after clipping or coiling within 96 h of rupture	Balloon angioplasty of bilateral A1, M1, P1, basilar, intradural vertebral artery, and supraclinoid ICA. Protocol later revised to exclude A1 and P1 (*N = *85)	No prophylactic balloon angioplasty (*N = *85)	Primary outcome: 3-month GOS Secondary outcome: delayed ischemic neurological deficit, TCD vasospasm, ICU, and hospital length of stay	Nonsignificant difference in delayed ischemic neurological deficits but less therapeutic angioplasty required in treatment group (*P = *0.03). No significant difference in GOS outcomes. LOS similar. Four patients had procedure related vessel perforation, three of whom died	Zwienenberg-Lee et al., Stroke 2008 [56]

Rho kinase inhibitor—fasudil						

Effect of AT877 on cerebral vasospasm after aneurysmal subarachnoid hemorrhage. Results of a prospective placebo-controlled double-blind trial	Randomized, placebo controlled, double-blind, multicenter study in Hunt Hess I–IV clipped SAH patients	Fasudil (AT877) 30 mg IV over 30 minutes, TID × 14 days (*N = *131)	Placebo (*N = *136)	Primary outcome: reduction of incidence or severity of angiographic vasospasm, reduction of incidence and size of low-density CT lesions due to vasospasm, reduction of incidence of symptomatic vasospasm, poor outcome (1-month GOS) due to vasospasm	Fasudil significantly reduced angiographic vasospasm (38% in treatment group versus 61% in placebo group, *P = *0.0023), infarcts reduced (16% in treatment versus 38% in placebo group, *P = *0.0013) and symptomatic vasospasm reduced (35% in treatment versus 50% in placebo, *P = *0.0247). Poor outcome (GOS 1–4) attributable to vasospasm occurred in 12% of treatment group and 26% of placebo group (*P = *0.0152). No serious adverse events in fasudil group	Shibuya et al., J Neurosurg 1992 [57]
Efficacy and safety of fasudil in patients with subarachnoid hemorrhage: final results of a randomized trial of fasudil versus nimodipine	Randomized, open label, multicenter study of SAH Hunt-Hess grade I–IV clipped patients	Fasudil 30 mg IV TID × 14 days (*N = *63)	Nimodipine 1-2 mg/h × 14 days (*N = *66)	Primary outcome: symptomatic vasospasm or infarct on HCT Secondary outcome: 1-month GOS	No difference in symptomatic vasospasm or HCT infarcts. Improved GOS outcomes in fasudil group (good outcome in 74.5% versus 61.7% in nimodipine group, *P = *0.040)	Zhao et al., Neurol Med Chir (Tokyo) 2011 [58]

Intensive insulin therapy						

The effect of intensive insulin therapy on infection rate, vasospasm, neurologic outcome and mortality in neurointensive care unit after intracranial aneurysm clipping in patients with acute subarachnoid hemorrhage: a randomized prospective Pilot trial	Randomized, controlled study	Intensive Insulin Infusion (80–120 mg/dL) × 14 d (*N = *40)	Conventional insulin infusion (glucose 80–220 mg/dL) × 14 d (*N* = 38)	Primary outcome: infection Secondary outcomes: vasospasm, 6-month mortality, and mRS	Higher infection rate in the conventional group (42% versus 27% in intensive group, *P* < 0.001). Similar vasospasm, mortality, and mRS at 6 months	Bilotta et al., J Neurosurg Anesthesiol 2007 [59]

Hypothermia						

Mild intraoperative hypothermia during surgery for intracranial aneurysm (IHAST)	Randomized, prospective, partially blinded, controlled, multicenter trial of WFNS grade I–III SAH patients	Intraoperative hypothermia (target 33°C with surface cooling) (*N = *499)	Intraoperative normothermia (target 36.5°C) (*N = *501)	Primary outcome: GOS at 90 days Secondary outcomes: 90-day mRS, Barthel Index, NIHSS, neuropsych testing, adverse events	No difference in 90 day GOS. Good GOS in 66% of hypothermia versus 63% of control patients (*P = *NS). No differences in death, length of stay, or discharge disposition. Postoperative bacteremia more common in the hypothermia group (5% versus 3%, *P = *0.05)	Todd et al., NEJM 2005 [60]

**Table 2:** Randomized controlled trials assessing neurologic outcomes after cardiac arrest—completed trials.

Trial name	Study design	Treatment group	Control group	Outcome measure	Results	Reference
Calcium channel blockers						

Effects of nimodipine on cerebral blood flow and cerebrospinal fluid pressure after cardiac arrest: correlation with neurologic outcome	Randomized, double-blind study	Nimodipine IV 0.25 mcg/kg/min (*N = *25)	Placebo (*N = *26)	Primary outcome: CBF measured by Xenon CT Secondary outcomes: ICP, neurological disability	Higher CBF in nimodipine group in first 4 hours after arrest (*P < *0.05) but no difference at 24 hours. No difference in neurological outcomes	Forsman et al., Anesth Analg 1989 [64]
Neuropsychological sequelae of cardiac arrest	Randomized, double-blind, placebo-controlled, study of out-of-hospital ventricular fibrillation	Nimodipine 10 mcg/kg IV then 0.5 mcg/kg/min × 24 hours (*N = *35)	Placebo (*N = *33)	Primary outcome: 3- and 12-month neuropsychological and cognitive batteries	No difference in neuropsychological or cognitive outcome between groups	Roine et al., JAMA 1992 [65]
A randomized clinical study of a calcium-entry blocker (lidoflazine) in the treatment of comatose survivors of cardiac arrest	Randomized, double-blind, placebo-controlled, multicenter study	Lidoflazine 1 mg/kg loading dose then 0.25 mg/kg at 8 and 16 hours after resuscitation (*N = *259)	Placebo (*N = *257)	Primary outcome: Pittsburgh Cerebral Performance Scale at 6 months Secondary outcomes: mortality, complications	No difference in 6-month neurological outcome or mortality between groups	Brain Resuscitation Clinical Trial II Study Group, NEJM 1991 [66]
Nimodipine after resuscitation from out-of-hospital ventricular fibrillation: a placebo-controlled, double-blind, randomized trial	Randomized, double-blind, placebo-controlled, study of out-of-hospital ventricular fibrillation	Nimodipine 10 mcg/kg IV then 0.5 mcg/kg/min × 24 hours (*N = *75)	Placebo (*N = *80)	Primary outcome: survival, 1-year GOS Secondary outcomes: death related to anoxic encephalopathy, GCS at 24 hours and 1 week, 3- and 12-month mini-mental state exam, activities of daily living, Barthel index, neurological exam, seizure, SPECT, myocardial infarction, arrhythmias	No difference in the survival rate, GOS at 3 or 12 months. No difference in minimental state exam, activities of daily living, or seizures	Roine et al., JAMA 1990 [67]

Neuroprotective						

Coenzyme Q 10 combined with mild hypothermia after cardiac arrest: a preliminary study	Randomized, placebo-controlled, double-blind, single-center study of out-of-hospital cardiac arrest	Hypothermia 35-36°C × 24 hours + Coenzyme Q 10 250 mg PO × 1 then 150 mg PO TID (*N = *25)	Hypothermia 35-36°C × 24 hours + Placebo (*N = *24)	Primary outcome: survival to ICU discharge Secondary outcomes: 3-month survival, 3-month GOS, S100 levels	3-month survival was 68% in the treatment group and 29% in the control group (*P = *0.0413). There was no significant difference in survival until discharge or GOS outcome	Damian et al., Circulation 2004 [68]

Thrombolytics						

A pilot randomized trial of thrombolysis in cardiac arrest (the TICA trial)	Randomized, double-blind, placebo controlled, single-center, feasibility trial for out-of-hospital cardiac arrest	Tenecteplase 50 mg IV × 1 (*N = *19)	Placebo (*N = *16)	Primary outcome: ROSC Secondary outcomes: survival to ED, ICU and hospital discharge	ROSC in 42% of tenecteplase and 6% of placebo group. No difference in survival to hospital discharge	Fatovich et al., Resuscitation 2004 [69]
Thrombolysis during resuscitation for out-of-hospital cardiac arrest	Randomized, double-blind, controlled, multicenter study of out-of-hospital cardiac arrest	Tenecteplase 0.5 mg/kg IV (*N = *525)	Placebo (*N = *525)	Primary outcome: survival at 30 days Secondary outcomes: survival to admission, ROSC, 24-hour survival, survival to discharge, cerebral performance score at discharge	No difference in 30-day survival, hospital admission, ROSC, 24-hour survival, discharge, or neurologic outcome. More intracranial hemorrhages in treatment group	Böttiger et al., NEJM 2008 [70]

Steroids and pressors						

Vasopressin, epinephrine, and corticosteroids for In-hospital cardiac arrest	Randomized, double-blind, placebo-controlled, single-center study	Vasopressin 20 IU IV + epinephrine 1 mg IV + methylprednisolone 40 mg IV followed by hydrocortisone (*N = *48)	Epinephrine + placebo (*N = *52)	Primary outcome: ROSC, survival to discharge Secondary outcomes: blood pressure after CPR, organ failure-free days, discharge Glasgow-Pittsburg cerebral performance scale	More ROSC in treatment group (81% versus 52%, *P = *0.003) and more survival to discharge (19% versus 4%, *P = *0.02)	Mentzelopoulos et al., Arch Intern Med 2009 [71]

Pressors						

A comparison of standard-dose and high-dose epinephrine in cardiac arrest outside the hospital	Randomized, double-blind, prospective, multi-center study	Epinephrine 0.2 mg/kg IV (*N = *648)	Epinephrine 0.02 mg/kg IV (*N = *632)	Primary outcome: return of spontaneous circulation (ROSC), admission to the hospital Secondary outcome: cerebral performance scale at admission, and discharge	No difference in ROSC rates, admission, survival, or discharge neurological status	Brown et al., NEJM 1992 [72]
Standard doses versus repeated high doses of epinephrine in cardiac arrest outside the hospital	Randomized, double-blind, prospective, single-center study	Repeated epinephrine 5 mg IV (*N = *271)	Repeated epinephrine 1 mg IV (*N = *265)	Primary outcome: ROSC Secondary outcomes: admission to the hospital, discharge, cerebral performance category at discharge and 6 months	No difference in ROSC, admission, discharge, or 6-month neurological outcomes	Choux et al., Resuscitation 1995 [73]
A randomized, double-blind comparison of methoxamine and epinephrine in human cardiopulmonary arrest	Randomized, double-blind, single-center study	Methoxamine 40 mg bolus IV then 40 mg 4 minutes later (*N = *77)	Epinephrine 2 mg bolus then 2 mg IV q 4 min (*N = *68)	Primary outcome: Mortality and Glasgow-Pittsburgh coma score Secondary outcomes: ROSC, successful resuscitation	No difference in ROSC or neurologic outcome, initial resuscitation, or survival to discharge	Patrick et al., Am J Respir Crit Care Med 1995 [74]
Randomised comparison of epinephrine and vasopressin in patients with out-of-hospital ventricular fibrillation	Randomized, double-blind, single-center, controlled study of out-of-hospital ventricular fibrillation patients who failed defibrillation	Vasopressin 40 IU IV (*N = *20)	Epinephrine 1 mg IV (*N = *20)	Primary outcome: survival to admission Secondary outcome: 24-hour survival, survival to discharge, GCS at discharge	No significant difference in survival to admission but more vasopressin patients survived 24 hours (60% versus 20%, *P = *0.02). No difference in survival to discharge or GCS at discharge	Lindner et al., Lancet 1997 [75]
High-dose versus standard-dose epinephrine treatment of cardiac arrest after failure of standard therapy	Randomized, controlled, single-blind, multicenter study of patients who had failed on standard dose of epinephrine 0.5–1.0 mg IV	Epinephrine 0.1 mg/kg IV up to 4 doses (*N = *78)	Epinephrine 0.01 mg/kg IV up to 4 doses (*N = *62)	Primary outcome: improvement in cardiac rhythm or ROSC Secondary outcomes: GCS at 6, 24, and 72 hours	No differences in ROSC, survival, or neurologic function between groups	Sherman et al., Pharmacotherarpy 1997 [76]
A comparison of repeated high doses and repeated standard doses of epinephrine for cardiac arrest outside the hospital	Randomized, controlled, prospective multicenter study	Epinephrine 5 mg IV up to 15 doses at 3-minute intervals (*N = *1677)	Epinephrine 1 mg IV up to 15 doses at 3-minute intervals (*M* = 1650)	Primary outcome: ROSC, admission to the hospital, number of admissions after a single dose of epinephrine, hospital discharge Secondary outcomes: survival, neurological outcome by GCS and cerebral performance scale	Significantly more ROSC in high dose group (40% versus 36% of control group, *P = *0.02) and more survival to admission (26.5% versus 23.6% of controls, *P = *0.05). No difference in survival to discharge or neurological status	Gueugniaud et al., NEJM 1998 [77]
Vasopressin versus epinephrine for in hospital cardiac arrest: a randomized controlled trial	Randomized, controlled, triple-blind, multicenter study of in-hospital cardiac arrest for asystole, PEA, or refractory ventricular fibrillation	Vasopressin 40 IU IV (first pressor) (*N = *104)	Epinephrine 1 mg IV (first pressor) (*N = *96)	Primary outcome: survival for 1 hour Secondary outcomes: survival to hospital discharge, modified mini-mental state exam at discharge, cerebral performance score at discharge, ROSC, adverse events	No difference in survival at 1 hour or survival to hospital discharge. No difference in mini-mental state exam scores or cerebral performance scores	Stiell et al., Lancet 2001 [78]
A comparison of vasopressin and epinephrine for out-of-hospital cardiopulmonary resuscitation	Randomized, controlled, multicenter study of out-of-hospital cardiac arrest with ventricular fibrillation failing defibrillation, PEA, or asystole	Vasopressin 40 IU IV × 2 doses maximum (*N = *589)	Epinephrine 1 mg IV × 2 doses maximum (*N = *597)	Primary outcome: survival to hospital admission Secondary outcomes: survival to hospital discharge, cerebral performance score in survivors	No difference in survival to admission among patients with ventricular fibrillation or PEA. Higher rates of hospital admission for asystole in vasopressin group (29% versus 20%, *P = *0.02) and hospital discharge (4.7% versus 1.5% with epinephrine, *P = *0.04). Patients who received rescue epinephrine after vasopressin had better survival to admission and discharge than the epinephrine alone group. No difference in cerebral performance	Wenzel et al., NEJM 2004 [79]
Vasopressin and epinephrine versus epinephrine alone in cardiopulmonary resuscitation	Randomized, controlled, multicenter trial	Epinephrine 1 mg IV + Vasopressin 40 IU IV (*N = *1442)	Epinephrine 1 mg IV + Placebo (*N = *1452)	Primary outcome: Survival to hospital admission Secondary outcomes: ROSC, survival to hospital discharge, good neurological recovery by cerebral performance scale, and GCS, 1-year survival	No differences in survival to admission, ROSC, survival to discharge, 1-year survival, or good neurologic recovery	Gueugniaud et al., NEJM 2008 [80]

Magnesium						

Randomised trial of magnesium in in-hospital cardiac arrest (MAGIC trial)	Randomized, placebo-controlled, single-center study of cardiac arrest in the ICU or general ward	Magnesium 2 g IV bolus then 8 g over 24 hours (*N = *76)	Placebo (*N = *80)	Primary outcome: ROSC Secondary outcomes: 24-hour survival, survival to hospital discharge, GCS, and discharge Karnofsky	No difference in ROSC, 24-hour survival, survival to discharge, or GCS	Thel et al., Lancet 1997 [81]
Magnesium in cardiac arrest (the MAGIC trial)	Randomized, double-blind, placebo-controlled, single-center study of out-of-hospital cardiac arrest	MgSO_4_ 5 g IV × 1 (*N = *31)	Placebo (*N = *36)	Primary outcome: ECG rhythm 2 minutes after drug, ROSC Secondary outcomes: survival to ED, ICU, and hospital discharge	No differences in ROSC or survival	Fatovich et al., Resuscitation 1997 [82]
Magnesium sulfate in the treatment of refractory ventricular fibrillation in the prehospital setting	Randomized, double-blind, placebo-controlled, multicenter study of prehospital ventricular fibrillation refractory to 3 shocks	MgSO_4_ 2 g IV × 1 (*N = *58)	Placebo (*N = *58)	Primary outcome: ROSC Secondary outcomes: admission to hospital, hospital discharge	No difference in ROSC, survival to admission or discharge	Allegra et al., Resuscitation 2001 [83]
A randomized trial to investigate the efficacy of magnesium sulphate for refractory ventricular fibrillation	Randomized, double-blind, placebo-controlled trial of ventricular fibrillation refractory to 3 shocks	MgSO_4_ 2–4 g IV × 1 (*N = *52)	Placebo (*N = *53)	Primary outcome: ROSC Secondary outcome: hospital discharge	No differences in ROSC or survival to discharge	Hassan et al., Emerg Med J 2002 [84]
Randomized clinical trial of magnesium, diazepam, or both after out-of-hospital cardiac arrest	Randomized, double-blind, placebo-controlled factorial design study	Tier 1: magnesium 2 g IV + placebo (*N = *75) Tier 2: diazepam 10 mg IV + placebo (*N = *75) Tier 3: magnesium 2 g IV + Diazepam 10 g IV (*N = *75)	Placebo only (*N = *75)	Primary outcome: awakening at 3 months (comprehensible speech and command following) Secondary outcome: days to awakening, days to death, independent at 3 months	No difference in neurological outcome between the 3 groups	Longstreth et al., Neurology 2002 [85]

Insulin						

Intravenous glucose after out-of-hospital cardiopulmonary arrest: a community-based randomized trial	Randomized, single-center controlled study	5% dextrose (D5W) infusion (*N = *374)	0.45 saline infusion (*N = *374)	Primary outcome: command following or comprehensible speech Secondary outcomes: survival to hospital admission and discharge	No difference in neurological outcomes, or survival to admission or discharge	Longstreth et al., Neurology 1993 [86]
Strict versus moderate glucose control after resuscitation from ventricular fibrillation	Randomized, controlled, multicenter study of out-of-hospital ventricular fibrillation cardiac arrest	Strict glucose control (4–6 mmol/L) with insulin infusion × 48 hours (*N = *39)	Moderate glucose control (6–8 mmol/L) with insulin infusion × 48 hours (*N = *51)	Primary outcome: 30-day all-cause mortality after ROSC Secondary outcomes: neuron-specific enolase levels at 24 and 48 hours	No difference in 30-day mortality	Oksanen et al., Intensive Care Med 2007 [87]

Hypothermia						

Treatment of comatose survivors of out-of-hospital cardiac arrest with induced hypothermia	Randomized, controlled, single-blind, prospective study	Hypothermia 33°C × 12 h (*N = *43)	Normothermia 37°C (*N = *34)	Primary outcome: discharge disposition Secondary outcomes: adverse events, hemodynamic parameters	Good discharge disposition in 49% of treatment group compared to 26% of normothermia group (*P = *0.046). No difference in adverse events	Bernard et al., NEJM 2002 [88]
Mild therapeutic hypothermia to improve the neurologic outcome after cardiac arrest	Randomized, controlled, single-blind, multicenter, prospective study	Hypothermia 32–34°C × 24 h (*N = *137)	Normothermia 37°C (*N = *138)	Primary outcome: 6 month neurologic outcome using Pittsburgh cerebral performance scale Secondary outcome: 6-month mortality, complications at 7 days	Hypothermia group had more favorable neurological outcome at 6 months (55% versus 39% of normothermia group, *P = *0.009). Less death in hypothermia group (41% versus 55% in normothermia group, *P = *0.02). No difference in complication rates	The hypothermia after cardiac arrest study group, NEJM 2002 [89]
Pilot randomized clinical trial of prehospital induction of mild hypothermia in out-of-hospital cardiac arrest patients with a rapid infusion of 4 degrees C normal saline	Randomized, controlled, safety and feasibility study of out-of-hospital cardiac arrest	2 L 4°C normal saline infusion (*N = *63)	Standard care (*N = *62)	Primary outcome: esophageal temperature, adverse events Secondary outcomes: awakening, hospital discharge	Significant differences in temperature between groups (*P < *0.001). No difference in awakening or hospital discharge	Kim et al., Circulation 2007 [90]
Prehospital therapeutic hypothermia for comatose survivors of cardiac arrest: a randomized controlled trial	Randomized controlled trail of out-of-hospital cardiac arrest	4°C Ringers solution 30 mL/kg to target temperature 33°C (*N = *19)	Conventional fluid therapy (*N = *18)	Primary outcome: nasopharyngeal temperature Secondary outcomes: hospital mortality and cerebral performance scale	Lower core temperature in the treatment group (*P < *0.001). No difference in safety, mortality, or neurologic outcome	KÄmÄrÄinen et al., Acta Anaesthesiol Scand 2009 [91]
Intra-arrest transnasal evaporative cooling: a randomized, prehospital, multicenter study (PRINCE: pre-rosc intranasal cooling effectiveness)	Randomized, controlled, prospective, single-blind, multicenter study for out-of-hospital arrest	Intra-arrest intranasal cooling with RhinoChill device + cooling at hospital arrival to 34°C (*N = *93)	Standard care with cooling at hospital arrival to 34°C (*N = *101)	Primary Outcome: adverse events, length of stay, mechanical ventilation days, ROSC, survival to discharge, discharge Pittsburgh cerebral performance scale.	Time to target temperature was shorter in the intranasal cooling group (*P = *0.03). No difference in ROSC, survival of admitted patients, or neurologic outcome at discharge	Castrén et al., Circulation 2010 [92]

Chest compressions						

A comparison of active compression-decompression cardiopulmonary resuscitation with standard cardiopulmonary resuscitation for cardiac arrests occurring in the hospital	Randomized, controlled, single center study	CPR using suction device (Ambu CardioPump) (*N = *29)	Standard CPR (*N = *33)	Primary outcome: ROSC Secondary outcomes: 24-hour survival, hospital discharge, GCS at 24 hours	ROSC occurred in 62% of treatment group versus 30% of control group (*P < *0.03) and 45% of treatment group survived 24 hours compared to 9% of control group (*P < *0.004). GCS at 24 hours was better in the treatment group (*P < *0.02)	Cohen et al., NEJM 1993 [93]
The Ontario Trial of Active Compression-Decompression Cardiopulmonary Resuscitation for In-Hospital and Prehospital Cardiac Arrest	Randomized, single-blind, multicenter controlled trial of prehospital and in-hospital cardiac arrest	Active compression-decompression CPR using a suction device (*N = *906)	Standard CPR (*N = *878)	Primary outcome: survival for 1 hour Secondary outcome: survival to hospital discharge, modified mini-mental state exam, cerebral performance scale	No differences in survival at 1 hour, survival until hospital discharge or mini-mental state exam for either prehospital or in-hospital arrest	Stiell et al., JAMA 1996 [94]
Cardiopulmonary resuscitation by chest compression alone or with mouth-to-mouth ventilation	Randomized controlled study of out-of-hospital cardiac arrest	Bystander chest compression plus mouth to mouth resuscitation (*N = *279)	Bystander chest compressions alone (*N = *241)	Primary outcome: survival to hospital discharge Secondary outcomes: admission to the hospital, neurological status	Similar outcome with bystander chest compressions alone versus chest compressions with mouth to mouth	Hallstrom et al., NEJM 2000 [95]
Constant flow insufflations of oxygen as the sole mode of ventilation during out-of-hospital cardiac arrest	Randomized, controlled study of out-of-hospital cardiac arrest	Constant flow insufflations of oxygen (*N = *487)	Standard endotracheal intubation and mechanical ventilation (*N = *457)	Primary outcome: survival to ICU discharge Secondary outcomes: ROSC, survival to hospital admission, spO_2_ > 70%	No difference in ROSC, hospital admission or ICU discharge. Higher O_2_ sats in continuous flow insufflation group	Bertrand et al., Intensive Care Med 2006 [96]
Compression-only CPR or standard CPR in out-of-hospital cardiac arrest	Randomized, controlled, multicenter study of out-of-hospital cardiac arrest	Compression only CPR (*N = *620)	Standard CPR (*N = *656)	Primary outcome: 30 day survival Secondary outcomes: 1 day survival, ROSC, survival to hospital discharge	Similar 30-day survival, 1-day survival and survival to hospital discharge	Svensson et al., NEJM 2010 [97]
CPR with chest compression alone or with rescue breathing	Randomized, controlled, multicenter study of out-of-hospital cardiac arrest	Chest compressions alone (*N = *981)	Chest Compressions + Rescue breathing (2 breaths to 15 chest compressions) (*N = *960)	Primary outcome: survival to hospital discharge Secondary outcomes: discharge cerebral performance Score, ROSC	No difference in survival to hospital discharge or in neurologic outcome. Trend toward improved survival at discharge in those with cardiac cause of arrest and shockable rhythm (*P = *0.09)	Rea et al., NEJM 2010 [98]
A trial of an impedance threshold device in out-of-hospital cardiac arrest	Randomized, controlled, double-blinded, multicenter study of out-of-hospital cardiac arrest	Impedance threshold device (ITD) which increasing negative intrathoracic pressure and improves cardiac output (*N = *4373)	Sham ITD (*N = *4345)	Primary outcome: survival to hospital discharge with mRS 0–3 Secondary outcomes: survival to ED admission, hospital admission and hospital discharge, adverse events	No difference in survival with good mRS	Aufderheide et al., NEJM 2011 [99]

Adenosine antagonist						

Aminophylline in bradyasystolic cardiac arrest: a randomized placebo-controlled trial	Randomized, placebo-controlled, double-blind, multicenter study of asystole and PEA arrest unresponsive to epinephrine and atropine	Aminophylline 250 mg (*N = *486)	Placebo (*N = *485)	Primary outcome: ROSC Secondary outcomes: duration of ROSC, survival to admission, survival to discharge, length of stay, 24-hour tachyarrhythmias, 24-hour seizures, 1-year neurologic outcome by GCS, Glasgow-Pittsburgh cerebral and overall performance scales, modified mini-mental state exam, functional status questionnaire	No difference in ROSC. More tachyarrhythmias in the treatment group. Survival to hospital admission and survival to discharge were not different	Abu-Laban et al., Lancet 2006 [100]

Fluid management						

Capillary leakage in postcardiac arrest survivors during therapeutic hypothermia: a prospective, randomized study	Randomized, controlled study	7.2% hypertonic saline with 6% poly starch solution × 24 hours (*N = *10)	Standard Fluid (Ringer's acetate) × 24 hours (*N = *9)	Primary outcome: amount of fluid administered in 24 hours Secondary outcome: MRI vasogenic edema	The treatment group required significantly less fluid than the control group. There was no difference in MRI brain edema	Heradstveit et al., Scand J Trauma Resusc Emerg Med 2010 [101]

Barbiturate						

Randomized clinical study of thiopental loading in comatose survivors of cardiac arrest	Randomized, controlled, multicenter study	Thiopental 30 mg/kg IV load (*N = *131)	Standard therapy (*N = *131)	Primary outcome: Pittsburgh cerebral Performance scale at 6 and 12 months Secondary outcomes: best neurological performance ever obtained during followup, time to recovery	No difference in neurological outcome or mortality between groups	Brain Resuscitation Clinical Trial I Study Group. NEJM 1986 [102]

Calcium						

Calcium chloride: reassessment of use in asystole	Randomized, double-blind, placebo-controlled study in prehospital asystolic cardiac arrest refractory to epinephrine, bicarbonate, and atropine	Calcium chloride (*N = *18)	Placebo (*N = *14)	Primary outcome: ROSC Secondary outcome: hospital discharge	No difference in ROSC	Stueven et al., Ann Emerg Med 1984 [103]
The effectiveness of calcium chloride in refractory electromechanical dissociation	Randomized, blinded, placebo-controlled study of prehospital PEA arrest refractory to epinephrine and bicarbonate	Calcium chloride (*N = *48)	Placebo (*N = *42)	Primary outcome: ROSC Secondary outcomes: survival to hospital discharge	No difference in ROSC but subgroup of patients with widened QRS did have more ROSC in calcium group (*P = *0.028)	Stueven et al., Ann Emerg Med 1985 [104]
Lack of effectiveness of calcium chloride in refractory asystole	Randomized, blinded, placebo controlled study of prehospital asystolic cardiac arrest refractory to epinephrine, bicarbonate, and atropine	Calcium chloride (*N = *39)	Placebo (*N = *34)	Primary outcome: ROSC Secondary outcomes: survival to hospital discharge	No difference in ROSC or hospital discharge	Stueven et al., Ann Emerg Med 1985 [105]

Sodium bicarbonate						

Buffer therapy during out-of-hospital cardiopulmonary resuscitation	Randomized, double-blind, placebo-controlled study of out-of-hospital asystole or ventricular fibrillation refractory to first defibrillation attempt	Sodium bicarbonate 250 mL IV × 1 (*N = *245)	Placebo (*N = *257)	Primary outcome: survival to ICU admission, survival to hospital discharge	No difference in survival to ICU admission or hospital discharge	Dybvik et al., Resuscitation 1995 [106]
Sodium bicarbonate improves outcome in prolonged prehospital cardiac arrest	Randomized, double-blind, placebo-controlled trial of prehospital cardiac arrest	Sodium bicarbonate (1 meq/kg) IV × 1 (*N = *175)	Placebo (*N = *155)	Primary outcome: survival to ED Secondary outcomes: ROSC	No difference in survival to ED admission or ROSC. Better survival with bicarbonate in the prolonged (>15 minute) arrest group (*P = *0.007)	Vukmir and Katz,Am J Emerg Med 2006 [107]

Hemofiltration						

High-volume hemofiltration after out-of-hospital cardiac arrest: a randomized study	Randomized, controlled trial of out-of-hospital ventricular fibrillation or asystole cardiac arrest	Tier 1: hemofiltration (200 mL/kg/h) over 8 hours (*N = *20) Tier 2: hemofiltration plus hypothermia to 32°C × 24 hours (*N = *22)	Standard care (*N = *19)	Primary outcome: survival at 6 months Secondary outcome: intractable shock, Pittsburgh cerebral performance scale	Significantly better survival compared to control in hemofiltration group (*P = *0.026) and hemofiltration plus hypothermia group (*P = *0.018). No difference in 6-month neurologic outcome	Laurent et al., J Am Coll Cardiol 2005 [108]

Rhythm analysis						

Early versus later rhythm analysis in patients with out-of-hospital cardiac arrest	Cluster randomized, controlled, multicenter study of out-of-hospital cardiac arrest	Early rhythm analysis: 30–60 seconds of EMS CPR followed by ECG analysis (*N = *5290)	Later rhythm analysis: 180 seconds of EMS CPR followed by ECG analysis (*N = *4643)	Primary outcome: survival to hospital discharge with mRS 0–3 Secondary outcomes: survival to discharge, survival to hospital admission, ROSC	No difference in outcome between a brief and longer period of CPR before ECG analysis of rhythm	Stiell et al., NEJM 2011 [109]

**Table 3:** Randomized controlled trials assessing neurologic outcomes after aneurysmal Subarachnoid hemorrhage—ongoing trials.

Trial name	Study design	Treatment group	Control group	Target enrollment	Outcome measure	PI	Comments
Statins							

Statins and cerebral blood flow in SAH	Randomized, double-blind efficacy study	Simvastatin 80 mg/d for 21 days	Placebo	60	Primary outcome: resting CBF and autoregulation 7–10 days after SAH Secondary outcomes: OEF and CMRO_2_ 7–10 days after SAH	Michael Diringer, NCT00795288	Uses PET to understand the mechanism of statin use in vasospasm
The role of statins in preventing cerebral vasospasm secondary to subarachnoid hemorrhage	Randomized, double-blind, parallel assignment	Simvastatin 80 mg PO qd × 21 days	Placebo	80	Primary outcome: 6-month clinical outcome	Eberval Figueiredo, NCT01346748	—
Use of simvastatin for the prevention of vasospasm in aneurysmal subarachnoid hemorrhage	Randomized, double-blind, parallel assignment efficacy trial	Tier 1: Simvastatin 40 mg × 21 d or Tier 2: Simvastatin 80 mg × 21 d	Placebo	150	Primary outcome: 21-day GOS, mRS, and Barthel Index Secondary outcome: clinical vasospasm	Ben Roitberg, NCT00487461	—
High-dose simvastatin for aneurysmal subarachnoid hemorrhage (HDS-SAH)	Randomized, parallel assignment, double-blind efficacy study	Simvastatin 80 mg PO × 21 days	Simvastatin 40 mg PO × 21 days	240	Primary outcome: delayed ischemic neurological deficit Secondary Outcomes: LFTs, rhabdomyolysis, 3-month mRS, cost effectiveness	George Wong, NCT01077206	There may be a biochemical and neuroprotective dose-related relationship between simvastatin and delayed ischemic neurological deficits.
Simvastatin in aneurysmal subarachnoid hemorrhage (STASH): a multicentre randomised controlled clinical trial	Randomized, placebo-controlled, double-blind phase III trial	Simvastatin 40 mg PO qd × 21 days	Placebo	1600	Primary outcome: 6-month mRS Secondary outcome: need and intensity of delayed ischemic deficit rescue therapy, incidence and duration of delayed ischemic deficits, incidence and severity of sepsis, length of stay, discharge disposition	Peter Kirkpatrick, NCT00731627	Simvastatin may improve CBF and inflammation following SAH

Aneurysm repair							

International subarachnoid aneurysm trial II comparing clinical outcomes of Surgical clipping and endovascular coiling for ruptured intracranial aneurysms not included in the original ISAT study (ISAT II)	Randomized, open label, safety/efficacy study of WFNS I–IV	Surgical Clipping	Endovascular Coiling	1724	Primary outcome: 12-month mRS > 2 Secondary outcomes: ICH following treatment, failure of aneurysm occlusion, all cause morbidity and mortality, aneurysm recurrence, hospitalization > 20 days or discharge other than home, aneurysm rebleed	Tim Darsaut, Max Findlay, and Jean Raymond, NCT01668563	ISAT included primarily small anterior circulation aneurysms. The optimal treatment of other locations and sizes of aneurysms remains unclear and coiling may not be as durable as clipping

Lipid peroxidation inhibitor							

Acetaminophen in aSAH to inhibit lipid peroxidation and cerebral vasospasm	Randomized, double-blind, placebo-controlled, safety/efficacy trial	Group1: Acetaminophen 1 g q 6 Group 2: NAC IV 0.5 g/h Group 3: Acetaminophen 1 g q6 + NAC 0.5 g/h Group 4: Acetaminophen 1.5 g q 6 + NAC 0.5 g/h	Placebo	120	Primary outcome: F2-IsoP biomarkers for lipid peroxidation. Secondary outcome: vasospasm and brain ischemia as assessed by CTA/CTP or MRI DWI	John Oates, NCT00585559	Hemoglobin released from lysed RBCs oxidizes and generates protein radicals that induce lipid peroxidation. Metabolites of peroxidations (F2-isoprostanes) are potent vasoconstrictors. Acetaminophen can inhibit these metabolites and NAC can inhibit lipid peroxidation

Neuroprotective drugs							

Effects of tiopronin on 3-aminopropanal level and neurologic outcome after aneurysmal SAH	Randomized, double-blind, phase 2 bioavailability	Tiopronin	Placebo	60	Primary outcome: serum and CSF 3AP levels Secondary outcomes: 12 month mRS, Barthel, Lawton, NIHSS, TICS adverse events	E Sander Connolly, NCT01095731	3AP is toxic metabolite produced during cerebral ischemia. It is neutralized by tiopronin
Lycopene following aneurysmal subarachnoid haemorrhage (LASH)	Randomized, double-blind, placebo-controlled, efficacy study	Lycopene 30 mg PO qd × 21 days	Placebo	124	Primary outcome: TCD vasospasm, duration of impaired autoregulation measured by TCD Secondary Outcomes: LDL, oxy-LDL, CRP, circulating endothelial cells, endothelial progenitor cells	Karol Budohoski, NCT00905931	Lycopene is a natural antioxidant that may reduce vascular injury and inflammation and limit vasospasm

Thrombolytics							

Intraventricular tPA in the management of aneurysmal subarachnoid hemorrhage	Randomized, placebo-controlled, double-blind safety trail	tPA intraventricular q 12 h × 5 doses	Placebo administered q 12 h × 5 doses	12	Primary outcome: HCT rate and variance of ventricular and cisternal clot clearance Secondary outcome: hemorrhagic complications, ventriculostomy-related infections, TCD vasospasm, CT angio vasospasm, symptomatic vasospasm, CSF cytokines and coagulation measurements, ICP, fever burden, volume of CSF drainage, 6-month GOSE and EuroQOL	Andreas Kramer, NCT01098890	Intraventricular TPA may accelerate clearance of IVH ameliorating vasospasm, hydrocephalus, and ICP

Transfusion							

Effect of red blood cell transfusion on brain metabolism in patients with SAH	Open label safety/efficacy study in SAH patients with Hgb < 12.5 g/dL and DCI, high risk for vasospasm or angiographic vasospasm	Transfusion of 1 unit of packed RBC over 1 hour	NA	48	Primary outcome: percent of brain regions with low oxygen delivery before and 1 hour after transfusion Secondary outcomes: relationship of oxygen delivery and angiographic vasospasm	Michael Diringer, NCT00968227	Uses PET to assess the relationship between Hct and oxygen delivery in SAH patients

Vasodilators							

Sildenafil for prevention of cerebral vasospasm (SIPCEVA)	Randomized, double-blind, placebo-controlled, safety and efficacy study	Tier 1: sildenafil 25 mg PO TID day 3–14 after SAH Tier 2: sildenafil 50 mg PO TID day 3–14 after SAH	Placebo	18	Primary outcome: New neurological deficit due to vasospasm up to 14 days after SAH Secondary outcomes: TCD spasm, mortality, adverse drug effects, length of stay, discharge mRS	Andre Cerutti Franciscatto, NCT01091870	—
Safety study of dantrolene in SAH	Randomized, double-blind safety study	Dantrolene	Placebo	30	Primary outcome: tolerability, hyponatremia Secondary outcomes: liver toxicity, hemodynamics, ICP, TCD, angiographic vasospasm treatments, 90-day GOS, mRS, and Barthel	Susanne Muehlschlegel, NCT01024972	Dantrolene is a muscle relaxant that may ameliorate vascular muscle tone and limit vasospasm
Safety and pharmacokinetic evaluation of nitrite for prevention of cerebral vasospasm	Randomized, single-blind, parallel assignment safety study	Tier 1: sodium nitrite 32 nmol/kg/min Tier 2: sodium nitrite 48 nmol/kg/min Tier 3: sodium nitrite 64 nmol/kg/min	Placebo vehicle	18	Primary outcome: pharmacokinetics of 14-day sodium nitrite infusion Secondary outcomes: safety and efficacy	Edward Oldfield, NCT00873015	—
Effects of prostacyclin infusion on cerebral vessels and metabolism in patients with subarachnoid hemorrhage	Randomized, placebo controlled, double-blind, parallel assignment, pharmacodynamics study	Tier 1: prostacyclin 1 ng/kg/min day 5–10 after SAH Tier 2: prostacyclin 2 ng/kg/min day 5–10 after SAH	Placebo, IV infusion day 5–10 after SAH	90	Primary outcome: vasospasm measured by CT perfusion Secondary outcomes: cerebral metabolism measured by microdialysis, 3-month GOS, clinical vasospasm, brain tissue oxygen, CT angio vasospasm, MAP, serum S100b	Rune Rasmussen, NCT01447095	Prostacyclin may cause vasodilation and ameliorate vasospasm

Hypertensive, hypervolemic therapy							

Induced hypertension for treatment of delayed cerebral ischemia after aneurysmal SAH HIMALAIA	Randomized, Single blind safety/efficacy study of patients with SAH and DCI (clinically defined)	Induced hypertension with vasopressors and fluids for 48 hours	No induced hypertension	240	Primary outcome: mRS at 3 months Secondary outcomes: proportion of treated patients who did not have clinical improvement of DCI symptoms within 24 hours, 30-day mortality, 3-month Barthel, SSQoL, hospital anxiety and depression scale, cognitive failures questionnaire, hospital complications, CTP results, medical costs	Arjen Slooter and Walter van den Bergh, NCT01613235	CBF measured in all patients using CTP at enrollment and 24–36 hours
Intensive management of pressure and volume expansion in patients with subarachnoid hemorrhage (IMPROVES)	Randomized, single-blind, factorial assignment	Tier 1: hypervolemia + conventional blood pressure Tier 2: normovolemia + hypertension Tier 3: hypervolemia + hypertension	Normal volume, normal blood pressure	20	Primary outcome: achievement of hemodynamic goals in each group	Miriam Treggiari, NCT01414894	Though triple H is a common therapy, its safety and efficacy have not been well quantified

CSF diversion							

EARLYDRAIN: outcome after early lumbar CSF: drainage in aneurysmal subarachnoid hemorrhage	Randomized, 2-arm controlled trial	Continuous lumbar CSF drainage of 120 mg qd × 7 d	Standard NICU care	300	Primary outcome: 6-month mRS Secondary outcomes: 6-month mortality, angiographic vasospasm, TCD vasospasm, shunt insertion rate at 6 months	Bardutzky J, NCT01258257	Lumbar drainage to remove blood from the basal cisterns may limit delayed cerebral ischemia
Cerebrospinal fluid (CSF) drainage study	Randomized, open label, parallel assignment study of SAH patients requiring external ventricular drainage (EVD)	High volume CSF diversion (EVD at 5 mmHg) × 10 days	Conventional CSF diversion (EVD at 15 mm Hg), weaned at physician discretion	20	Primary outcome: 90-day mRS Secondary Outcome: radiologic infarction, TCD or angiographic vasospasm, shunt placement, ventriculitis, discharge mRS, 90 day mini-mental status exam, length of stay	Giuseppe Lanzino, NCT01420978	More aggressive CSF drainage may improve brain microcirculation and perfusion and lead to better neurological outcomes

Antiepileptics							

Comparison of short duration levetiracetam to extended course for seizure prophylaxis after subarachnoid hemorrhage	Randomized, prospective, open label, parallel assignment, phase III, safety/efficacy study	Levetiracetam 1000 mg BID × 3 days	Levetiracetam 1000 mg BID × hospital stay	460	Primary outcome: In hospital seizures Secondary outcome: Incidence of seizure after hospital discharge, adverse drug reactions, length of stay, cognitive and functional outcomes	Rajat Dhar, NCT01137110	Antiepileptics can have long-term cognitive side effects. A short course may be just as efficacious as prolonged use
Antiepileptic drugs and vascular risk markers	Randomized, open label, parallel assignment study	Tier 1: phenytoin 5 mg/kg/d divided in 2 doses Tier 2: valproate 15 mg/kg/d divided in 3 doses Tier 3: levetiracetam 1000-1500 mg/d divided in 2 doses	No drug intervention	200	Primary outcome: serum cholesterol, non-HDL cholesterol, HDL, lipoprotein a, CRP Secondary outcome: acute seizures, late seizures, mRS at 8 and 16 weeks	Prema Kishna and Scott Mintzer, NCT00774306	Certain seizure medications may raise cholesterol levels and increase the risk of heart attack and stroke

Sedation							

Effects of dexmedetomidine on inflammatory cytokines in patients with aneurysmal subarachnoid hemorrhage	Randomized, open label, parallel assignment efficacy study	Dexmedetomidine 0.2–1.5 mcg/kg/h	Propofol 5–80 mcg/kg/min	10	Primary outcome: serum and CSF cytokines over 48 hours Secondary outcomes: sedative and analgesic requirements, RASS and CAM-ICU scores, length of stay, delayed cerebral ischemia, GOSE at discharge	Shaun Keegan and Brittany Woolf, NCT01565590	Dexmedetomidine may cause less inflammation over time than propofol

Rehabilitation							

Rehabilitation of patients after subarachnoid hemorrhage	Nonrandomized, open label, parallel assignment	Early multidisciplinary rehab and mobilization	No intervention	160	Primary outcome: 10-week GOS Secondary outcome: 3–6 month and 12-month GOSE, functional independence measure, coma recovery scale, disability rating scale, High-level Mobility Assessment tool, pain score	Tanja Karic and Angelika Sorteberg, NCT01656317	Early rehab may reduce complications and improve physical and cognitive function after SAH

Blood pressure control							

Safety and Efficacy Study of Clevidipine to Control Hypertension in Patients Admitted with Aneurysmal Subarachnoid Hemorrhage (CLASH)	Open label, safety, efficacy study, single group assignment (Phase 2)	Clevidipine IV 2–32 mg/h for 24–48 hours	NA	20	Primary: Blood pressure within target range	Panayiotis Varelas, NCT00978822	To assess how rapidly and safely Clevidipine can be used to control blood pressure in SAH patients.

Other							

Cervical spinal cord stimulation for the prevention of cerebral vasospasm	Nonrandomized, open label	Spinal cord stimulation using MTS Trial System 3510	NA	12	Primary outcome: cerebral vasospasm Secondary outcome: adverse events	Konstantin Slavin, NCT00766844	—

**Table 4:** Randomized controlled trials assessing neurologic outcomes after cardiac arrest—ongoing trials.

Trial name	Study design	Treatment group	Control group	Target enrollment	Outcome measure	PI	Comments
Neuroprotective drugs							

Selenium to Improve Neurological Outcome after Cardiac Arrest (SCPR)	Randomized, double-blind, placebo-controlled, single-center, phase 2a efficacy study	Sodium-selenite infusion × 7 days	Placebo	52	Primary outcome: neuron-specific enolase Secondary outcomes: inflammation and oxidative stress markers, NIHSS and Glasgow Pittsburgh performance score at 6 months, selenium blood levels, glutathione peroxidase plasma levels	Vanessa Stadlbauer and Karlheinz Smolle, NCT01390506	Selenium can reduce oxidative stress after cardiac arrest and reduce inflammation

Magnesium							

Clinical Study of the LRS ThermoSuit System in Post Arrest Patients with Intravenous Infusion of Magnesium Sulfate	Randomized, double-blind, parallel assignment, safety/efficacy study of any rhythm	Thermosuit to target 34°C plus magnesium sulfate IV (30 mg/kg over 15 minutes)	ThermoSuit to target 34°C plus placebo (normal saline)	14	Primary outcome: cooling rate Secondary outcomes: time to target temperature, percentage of time in target temperature range, shivering, length of stay, neurologic status at discharge and 6 months, adverse events, survival at 24 hours, discharge and 30 days	Michael Holzer and Andreas Janata, NCT00593164	Tests new device to achieve therapeutic hypothermia and the impact of magnesium on cooling performance and hemodynamics

Hypothermia							

Target Temperature Management after Cardiac Arrest (TTM)	Randomized, double-blind, parallel assignment, multicenter, safety/efficacy trial for out-of-hospital cardiac arrest	Target temperature 36°C × 24 h	Target temperature 33°C × 24 h	850	Primary outcome: All cause mortality Secondary outcomes: 6-month composite all cause mortality and poor neurological outcome by cerebral performance scale, bleeding, 6-month neurological status and quality of life, mRS, Cerebral performance scale, mini-mental test, IQCODE, SF-36, adverse events	Niklas Nielsen and Hans, Friberg NCT01020916	Attempts to identify optimal hypothermia target temperature
Hypothermia After in-Hospital Cardiac Arrest (HACAinhospital)	Randomized, single-blind, parallel assignment, single-center, safety/efficacy study for in-hospital arrests of any rhythm	Mild therapeutic hypothermia 32–34°C × 24 hours.	Standard care, no hypothermia	440	Primary outcome: all cause mortality at 6 months Secondary outcomes: 6 month Glasgow-Pittsburgh cerebral performance scale, in-hospital all-cause mortality	Sebastian Wolfrum and Volkhard Kurowski, NCT00457431	Tests whether hypothermia treatment will improve outcome after in-hospital arrest of any rhythm
Intra-arrest Therapeutic Hypothermia in Prehospital Cardiac Arrest (HITUPPAC-BIO)	Randomized, open label, parallel assignment, efficacy trial	Hypothermia induction prehospital	Hypothermia induction at hospital arrival	250	Primary outcomes: brain injury biomarkers at 72 h Secondary outcomes: ROSC, survival 72 h, GCS at 48 hours, cerebral performance scale at 28 days	Guillaume Debaty Jean Francois Timsit, NCT00886184	Assess utility of early hypothermia prehospital
Induction of Mild Hypothermia Following Out-of-hospital Cardiac Arrest	Randomized, open label, single group assignment, efficacy study of any rhythm out of hospital arrest	Rapid infusion of 2 L of 4°C normal saline prior to ED arrival	Standard therapy	1364	Primary outcome: awake and command following at hospital discharge Secondary outcomes: days to awakening, days to death, 3 month neurological outcome	Francis Kim, NCT00391469	Tests whether rapid induction of hypothermia with cold saline infusion is efficacious
Comparing Therapeutic Hypothermia Using External and Internal Cooling for Post-Cardiac Arrest Patients	Randomized, open label, parallel assignment, efficacy trial	External device (Arctic Sun) induced hypothermia	Internal device (Alsius) induced hypothermia	51	Primary outcome: Survival to hospital discharge Secondary outcome: 1 year neurological status	Marcus Ong, NCT00827957	Identifying the most efficient method of cooling may improve outcome after cardiac arrest

Hypothermia + ECMO							

Refractory Out-of-Hospital Cardiac Arrest Treated with Mechanical CPR, Hypothermia, ECMO and Early Reperfusion (CHEER)	Nonrandomized, single group, open label, safety/efficacy trail for patients who fail standard resuscitation	Automated CPR, ECMO, coronary angiography, therapeutic hypothermia	NA	24	Primary outcome: survival to hospital discharge Secondary outcomes: cerebral performance Scale, time to ECMO insertion, neurologic biomarkers, cardiac recovery	Stephen Bernard and Dion Stub, NCT01186614	Aggressive resuscitation may improve outcome in patients who fail standard resuscitation
Hyperinvasive approach to out-of-hospital cardiac arrest using mechanical chest compression device, prehospital intraarrest cooling, extracorporeal Life support and early Invasive assessment compared to standard of care: Prague OHCA Study	Randomized, open-label, parallel group, safety/efficacy study	Prehospital mechanical compression device, intraarrest cooling and in hospital ECLS (compression device, Rhinochill, PLS ECMO)	Standard care	170	Primary outcome: composite endpoint of survival with good neurological outcome (cerebral performance scale) Secondary outcome: 30 day cerebral performance scale, 30 day cardiac recovery	Jan Belohlavek and Ondrej Smid, NCT01511666	Aggressive, early intervention may improve cerebral outcomes
Emergency Preservation and Resuscitation (EPR) for Cardiac Arrest from Trauma (EPR-CAT)	Nonrandomized, open label, parallel assignment, safety/efficacy study	Profound hypothermia < 10°C with cold saline infusion into aorta followed by resuscitation/rewarming with cardiopulmonary bypass	Standard treatment	20	Primary outcome: survival to hospital discharge without major disability by GOSE Secondary outcomes: achieving target temperature in 1 hour, 28 day survival, 6 month neurological function, multiple organ system dysfunction	Samuel Tisherman, NCT01042015	Resuscitation technique for trauma patients that have arrested from exsanguination

HypotherMia + xenon							

Effect of xenon and therapeutic hypothemia on brain and on neuroloigcal outcome following brain ischemia in cardiac arrest patients (Xe-hypotheca)	Randomzied, open label, parallel assignment, phase 2 safety/efficacy trial for ventricular fibrillation and nonperfusing ventricular tachycardia	Hypothermia 33°C × 24 h and Xenon inhalation × 24 hours target end tidal 40%	Hypothermia 33°C × 24 h	110	Primary outcome: PET and MRI ischemia at 24 hours and 10 days Secondary outcomes: neurological outcome at 6 months, TTE	Timo Laitio, NCT00879892	Xenon may be synergistically neuroprotective in combination with hypothermia post arrest by limiting cerebral hypoxia, neuronal loss, and mitochondrial dysfunction

Chest compresions							

Continuous chest compressions	Randomized, open label, multicenter, crossover assignment study of out-of-hospital cardiac arrest of any rhythm	Continuous chest compressions	Interrupted chest compressions with ventilation 30 : 2	23600	Primary outcome: survival to hospital discharge Secondary outcomes: mRS at discharge, adverse events	Myron Weisfeldt, NCT01372748	Continuous CPR without interruption for ventilation may be superior to interrupted compression with ventilation ratio of 30 : 2
LUCAS chest compressor versus manual chest compression in out-of-hospital sudden cardiac arrest: LUCAT trial	Randomized, open label, parallel assignment, efficacy study	Mechanical continuous chest compressions performed by LUCAS device	Manual chest compressions	400	Primary outcome: survival to hospital admission, survival to discharge with good neurological state by cerebral performance Scale Secondary outcomes: ROSC, end tidal CO_2_, SOFA scale, length of stay, metabolic and inflammatory markers, LV function	Francesc Carmona Jimenez, Rosa-Maria Lidon, NCT01521208	Mechanical chest compression may be superior to manual chest compression

Cerebral oxygenation							

Cerebral Oxygenation in Cardiac Arrest and Hypothermia	Open label, safety and efficacy study	Near-infrared monitoring	Standard therapy, no monitoring	70	Primary outcome: survival to discharge Secondary outcomes: cerebral performance scale at discharge and 12 months	Christian Storm, NCT01531426	Near-infrared spectroscopy (NIRS) could be a new-noninvasive marker for outcome after cardiac arrest. Low NIRS may correlate with poor outcome

**Table 5:** Number of randomized, controlled trials published and ongoing for aneurysmal subarachnoid hemorrhage and cardiac arrest.

Intervention	SAH		Cardiac arrest	
	Published *N* (%)	Ongoing *N* (%)	Published *N* (%)	Ongoing *N* (%)
Calcium channel blockers	10 (18)	0	4 (9)	0
Antifibrinolytics	5 (9)	0	0	0
Neuroprotective drugs	5 (9)	2 (8)	1 (2)	1 (7)
Statins	4 (7)	5 (20)	0	0
Aneurysm clip or coil	4 (7)	1 (4)	NA	NA
Lipid peroxidation inhibitor	4 (7)	1 (4)	0	0
Thrombolytics	3 (5)	1 (4)	2 (4)	0
Antiplatelets	3 (5)	0	0	0
Steroids	3 (5)	0	1 (2)	0
Transfusion/blood products/erythropoietin	3 (5)	1 (4)	0	0
Vasodilators	3 (5)	4 (16)	0	0
Pressors or HHH	2 (4)	2 (8)	9 (19.5)	0
Magnesium	2 (4)	0	5 (11)	1 (7)
Rho-kinase inhibitor (fasudil)	2 (4)	0	0	0
Adrenergic blockade	1 (2)	0	0	0
Endovascular therapy	1 (2)	0	NA	NA
Insulin/glucose control	1 (2)	0	2 (4)	0
Hypothermia	1 (2)	0	5 (11)	9 (64)
CSF diversion	0	2 (8)	0	0
Antiepileptics	0	2 (8)	0	0
Sedation	0	1 (4)	0	0
Rehabilitation	0	1 (4)	0	0
Blood pressure	0	1 (4)	0	0
Other	0	1 (4)	0	0
Chest compressions	NA	NA	7 (15)	2 (14)
Adenosine antagonist	0	0	1 (2)	0
Fluid management	0	0	1 (2)	0
Barbiturate	0	0	1 (2)	0
Cerebral oxygenation	0	0	0	1 (7)
Calcium chloride	0	0	3 (7)	0
Sodium bicarbonate	0	0	2 (4)	0
Hemofiltration	0	0	1 (2)	0
Rhythm analysis	0	0	1 (2)	0

Total	57	25	46	14
